# Specific alien plant species predominantly deliver nectar sugar and pollen but are not preferentially visited by wild pollinating insects in suburban riparian ecosystems

**DOI:** 10.1002/ece3.10441

**Published:** 2023-08-22

**Authors:** Chika Egawa, Teru Yuta, Asuka Koyama

**Affiliations:** ^1^ Institute for Agro‐Environmental Sciences National Agriculture and Food Research Organization Ibaraki Japan; ^2^ Yamashina Institute for Ornithology Chiba Japan; ^3^ Forestry and Forest Products Research Institute Ibaraki Japan

**Keywords:** biological invasion, floral diversity, floral resources, pollinator conservation, urban–rural boundary

## Abstract

The invasion of alien plants has been rapidly proceeding worldwide due to urbanisation. This might be beneficial to wild pollinating insects, since some alien plant species have large flowers and/or long flowering periods, which can increase nectar sugar and pollen availability. To determine the relative contribution of alien plants to floral resource supply and whether resource‐rich alien plants, if any, serve as an important food source of pollinating insects, we performed year‐round field observations in suburban riverbanks. We quantified the per‐unit‐area availability of nectar sugar and pollen delivered by alien and native flowering species and counted wild flower visitors (bees and wasps, hoverflies and butterflies) per plant species. The available nectar sugar and pollen per area were predominantly delivered by a few specific alien species, and the relative contribution of other species to floral resource provision was low throughout the period that wild flower visitors were observed. Nonetheless, the resource‐rich alien plants were not visited by as many insects as expected based on their contribution to resource provision. Rather, on a yearly basis, these plants received equal or even fewer visits than other flowering species, including resource‐poor natives. We show that despite their great contribution to the gross floral resource supply, resource‐rich alien plants do not serve as a principal food source for wild pollinating insects, and other plants, especially natives, are still needed to satisfy insect demand. For the conservation of pollinating insects in suburban ecosystems, maintaining floral resource diversity would be more beneficial than having an increase in gross floral resources by allowing the dominance of specific alien plants.

## Graphical abstract

We show that despite the great contribution to gross floral resource supply, resource‐rich alien plants do not serve as a principal food source of wild pollinating insects, and other plants, especially natives, are still needed to satisfy insect demand in suburban riparian ecosystems. For the conservation of pollinator insects, maintaining floral resource diversity would be more beneficial than having an increase in gross floral resources by allowing the dominance of specific alien plants.

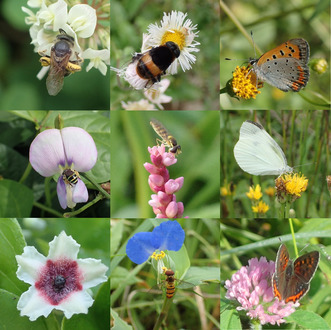

## 1 INTRODUCTION

Human land‐use change has drastically reduced natural vegetation that provides nectar sugar and pollen, primary floral resources, to wild pollinating insects (Baude et al., 2016; Carvell et al., 2006); therefore, it is considered a major cause of ongoing pollinator declines (Potts et al., 2010). On the other hand, land development has brought novel floral resources to the surrounding environment by promoting the invasion of alien plant species derived from gardening, farming and landscaping (Koyama et al., 2018; Tew et al., 2021; Wenzel et al., 2020). Alien plants usually become well integrated in pollination networks soon after their establishment (Kovács‐Hostyánszki, Piross, & Shebl, 2022; Seitz et al., 2020; Urbanowicz et al., 2020). However, the significance of alien plants in meeting the resource demand of wild pollinators has not yet been fully understood. Characterising floral resource provision by alien plants and the responses of wild pollinators to that resource supply will lead to a better understanding of the roles of alien plants and the entire impacts of land development on wild pollinators and pollination services.

Alien plant species sometimes have flowering characteristics distinct from those of native species, such as larger flowers and longer flowering periods (Bjerknes et al., 2007; Ghazoul, 2002; Pyšek & Richardson, 2007). These traits, combined with increased floral abundance, can make these plants a rich, months‐long source of nectar sugar and pollen (Hicks et al., 2016; Ortiz et al., 2021). This leads to an expectation that some, if not all, alien plant species would play a key role in floral resource supply in invaded ecosystems. In fact, from year‐round sampling, Tew et al. (2021) demonstrated that available nectar sugar is predominantly derived from alien ornamental plants in urban settings. Given that plants with greater floral rewards often attract more pollinators (Ferrero et al., 2013; Goodell & Parker, 2017; Makino & Sakai, 2007), a hypothesis on the roles of resource‐rich alien plants emerges; in invaded ecosystems, (1) resource‐rich alien plants are most frequently visited by wild pollinators as a principal food source, thereby largely contributing to meeting resource demand from pollinators. On the other hand, several studies have highlighted that floral resource diversity is the key to satisfying pollinating insects, for reasons including nutritious advantages of a dietary mix containing various plant species in promoting insect population growth (Behmer, 2009; Blüthgen & Klein, 2011). The findings raise an alternative hypothesis that (2) despite their high productivity, resource‐rich alien plants are only valued and visited by wild pollinators to a similar extent as other relatively resource‐poor plants and thus have little advantage in meeting pollinator resource demand. If this is the case, then the establishment of resource‐rich alien plants might have negative rather than positive consequences on wild pollinators, as they may outcompete other plant species and reduce floral resource diversity (Kovács‐Hostyánszki, Szigeti, et al., 2022). Verifying these hypotheses would provide both scientific and practical insights. However, few empirical tests have been conducted, especially in spontaneously established plant communities containing various alien and native species.

In this study, we tested the applicability of the hypotheses on the roles of alien plants by investigating floral resource provision and flower visits by insects in spontaneously established suburban plant communities. Specifically, we quantified the area‐based availability of nectar sugar and pollen derived from alien and native flowering species and counted insect visits to each flowering species in riverbanks at the urban–rural boundary in Ibaraki, Japan. Plant communities established on riverbanks surrounded by urban and agricultural areas are generally invaded by various alien plant species (Aronson et al., 2017). Additionally, riparian sites are known to act as important feeding grounds for a variety of wild pollinating insects, such as bees, hoverflies and butterflies (Cole et al., 2015; Verboven et al., 2014). Therefore, riparian plant communities at the urban–rural boundary provide an ideal setting for the study. To cover the entire period of flowering for all the established plant species and of the activity of flower‐visiting insects, we conducted year‐round field observations for two consecutive years. The year‐round observations allowed us to not only characterise seasonal fluctuations in floral resource provision and visitation by wild insects but also obtain comprehensive measures of relative species contributions to and diversity in available floral resources and insect‐visited flowers on a yearly basis.

## 2 MATERIALS AND METHODS

### 2.1 Field observations of floral abundance and insect visits

#### 2.1.1 Study sites

Field observations were conducted in plant communities spontaneously established on riverbanks of the Sakura River with a basin area of 350 km^2^ (36°09′40.32″ N, 140°05′04.63″ E), the Hanamuro River with a basin area of 39 km^2^ (36°04′33.53″ N, 140°08′04.74″ E) and two locations along the Kokai River with a basin area of 1043 km^2^ (coded Kokai 1: 36°12′40.54″ N, 140°00′25.63″ E and Kokai 2: 36°13′33.10″ N, 139°59′57.34″ E) in Ibaraki Prefecture, eastern Japan. At all sites, herbaceous communities were maintained by mowing as vegetation management (once a year at most). All sites are located at the urban–rural boundary, surrounded by farmland, fallow fields, forests and human infrastructure such as roadways. Although the two Kokai sites were along the same river, they were located at completely different points along a large riverbank. The distances between each site ranged from 2 to 13 km, which exceeds the foraging range of most species of bees (Rands & Whitney, 2011). Additionally, each site was separated by forests and human infrastructure. Based on these settings, we assumed that each site did not share pollinator populations, and we treated the four sites as independent replications and assessed whether there were common patterns among sites. The daily mean temperature in the area during the study period averaged 15.4°C, ranging from 4.2°C in January to 27.2°C in August (Japan Meteorological Agency Data: https://www.data.jma.go.jp/obd/stats/etrn/index.php, last accessed on 23 March 2023).

#### 2.1.2 Observation methods

**FIGURE 1:**
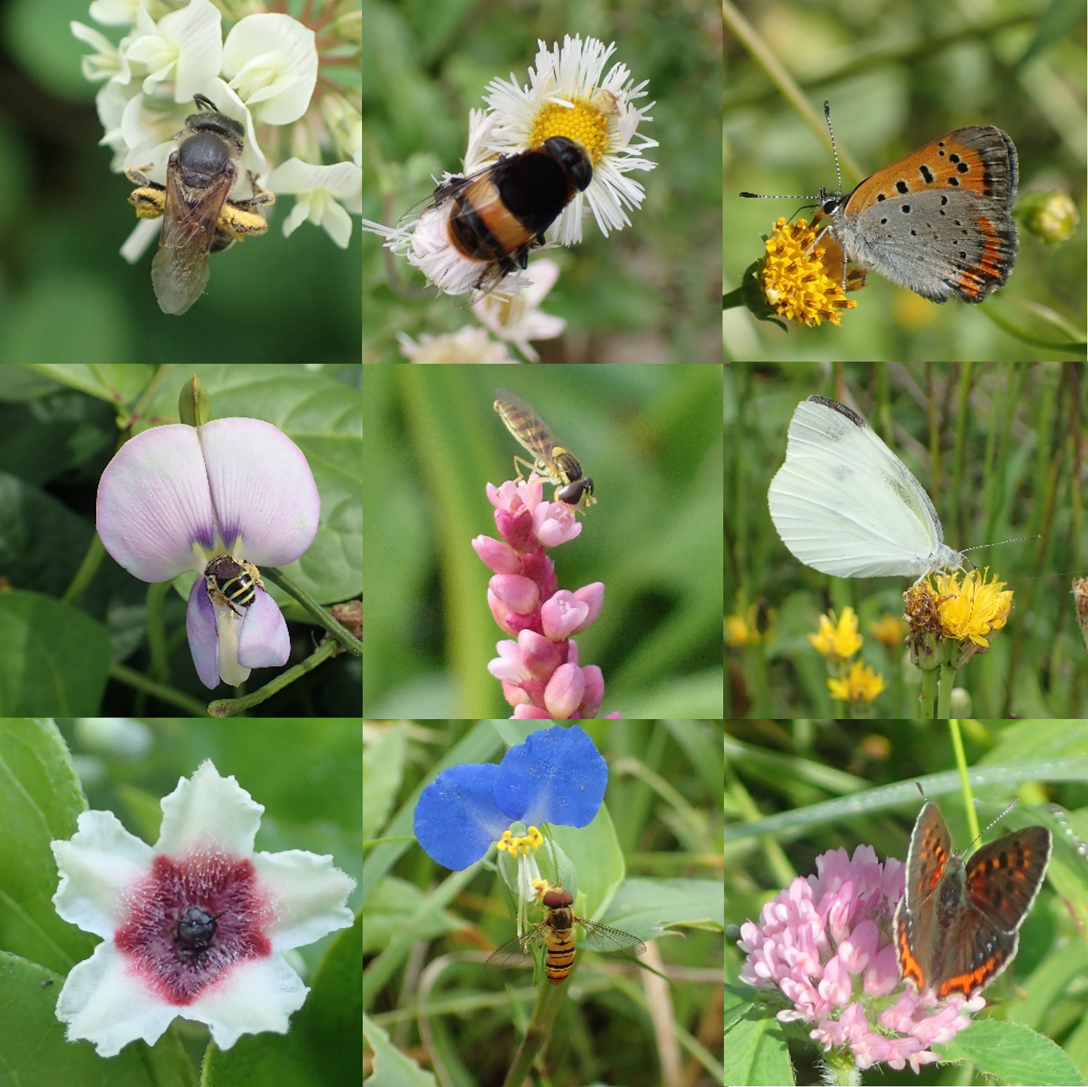
Photos of flower‐visiting insects touching the reproductive structures of plant species recorded in the study.

The observations were conducted from 21 April 2018 to 4 April 2020. As the main floral sources for pollinator insects, we focused on entomophilous species and excluded anemophilous species such as grasses and sedges from the investigation targets. We visited each of the four sites at 2‐week intervals throughout the first year from 21 April 2018 to 13 April 2019 and 1‐month intervals throughout the second year from 14 April 2019 to 4 April 2020. At Kokai 1, field observations were not conducted in November and December 2018 due to mowing management. For the same reason, field observations started on 28 May 2018 and ended on 12 August 2019 at Kokai 2. In both study years, observations of floral abundance and insect visits were performed in a set during the daytime (between 5:00 and 17:00) on sunny, non‐windy days. On each survey occasion, five 1 m^2^ plots were established at 5 m intervals along a 30 m transect placed along the rivers. In each plot, we counted the number of open floral units (defined as flower heads for Asteraceae and *Trifolium* (Fabaceae) and individual flowers for other species) for each of all flowering species. In total, we recorded 188,723 open floral units of 79 entomophilous species throughout 2 years of observation (plant species nomenclature was based on Ylist: http://ylist.info, last accessed on 22 March 2023). Following the measurement of floral abundance, flower visits by insects were observed for 10 min per plot by a single observer, recording the number of insects touching the reproductive structures of each plant species (Figure 1). We repeated this procedure once more with a different observer after all five plots were observed for the first round of 10 min. Therefore, in total, each plot was observed for 20 min per survey occasion. The observation hours (between 5:00 and 17:00) at each site were changed every census to account for variations in active time among insects. Because it was often unidentifiable whether each insect was collecting nectar, pollen or both, no distinction was made between flower visitors collecting different resources. The recorded insects were categorised into three major pollinator groups, that is, wild bees and wasps (Hymenoptera: Apoidea, Scolioidea and Vespoidea), hoverflies (Diptera: Syrphidae) and butterflies (Lepidoptera), and the number of insects was tallied by group. In this study, we define ‘wild’ insects as those that are not managed or domesticated by humans. We did not further identify individual insect species because our primary focus was to clarify the values of resource‐rich alien plants to wild pollinating insects overall. During the observation, we also recorded visits by Coleoptera, Hemiptera and European honeybees (*Apis mellifera*). Although Coleoptera and Hemiptera are also associated with pollination (Rader et al., 2016), we excluded these taxa from further analyses due to their small numbers. Similarly, we did not include data on European honeybees in the analyses because it is an artificially managed species. In addition, European honeybees are often kept by migratory beekeepers who constantly move around Japan with their bee colonies (Kamo et al., 2018; Okubo et al., 2021), and thus there may be artificial influences on their flower‐visiting timing.

### 2.2 Quantification of nectar sugar and pollen per flower and per plot area

We quantified floral resources supplied by each plant species in terms of nectar sugar mass and pollen volume, both of which are known as essential features to be considered in pollinator dietary studies (Buchmann & O'Rourke, 1991; Hicks et al., 2016). We collected flower samples in the observation sites during the study period, and measured nectar sugar mass and pollen volume based on Baude et al. (2016) and Hicks et al. (2016), although we applied a different nectar accumulation duration from these studies as outlined below and in Supporting Information S1. Quantification was conducted at the level of a single flower for all species except for Asteraceae and *Trifolium*, where quantification was conducted at the flower head level, in accordance with the floral units recorded in the field observations. Hereafter, ‘flower’ refers to the unit of quantification applied to each species (synonymous with floral unit). We confirmed the validity of the calculated values of nectar sugar mass and pollen volume by comparing our data with the data from Baude et al. (2016) and Hicks et al. (2016; for details, see Supporting Information S1).

#### 2.2.1 Measurement of nectar sugar mass per flower

Between 6:00 and 16:30 on rainless days, we randomly collected an average of 18 (range 5–53) open but not yet senescing flower samples from 56 out of 74 flowering species with nectaries recorded in the field survey on floral abundance (for the species list, see Table S1). We also collected flowers of six species that were not recorded in the field survey but appeared outside observation plots; the data were not used for analyses but were provided in Table S1 with the aim of making all relevant data available. Prior to sampling, the flowers were bagged with a fine net to avoid insect visits and were allowed to accumulate nectar for 2 h to ensure a measurable amount of nectar. The duration of nectar accumulation was shorter than that in previous studies (i.e. 24 h in Baude et al., 2016; Hicks et al., 2016; Tew et al., 2021) and would not be appropriate for assessing the absolute nectar productivity of species; however, because our focus was on capturing the relative differences among plant species in contribution to available floral resources, this accumulation duration can be justified for this study. Nectar was extracted using 0.25, 0.5, 1.0, 2.0, 5.0 or 10.0 μL microcapillary tubes (Drummond Microcaps) depending on the size of the flowers. The sugar concentration of the sampled nectar was then immediately measured using a hand‐held sucrose refractometer modified for small sample volumes (Bellingham and Stanley Eclipse 45‐81 and 45‐82). When the direct extraction of nectar was not possible due to the small size of flowers, we rinsed the flower with 2 μL of distilled water applied at the location of the nectaries using a micropipette and then measured the sugar concentration of the resulting solution after waiting for 1 min, following Baude et al. (2016) and Hicks et al. (2016). We calculated the sugar mass (*s*; μg) with the equation *s* = 10*dv*C, where *v* is the volume of nectar (μL), and *d* is the density of a sucrose solution with a concentration of C (g sucrose per 100 g solution) as read on the refractometer. The density of the sucrose solution was calculated as *d* = 0.0037921C + 0.0000178C^2^ + 0.9988603 (Baude et al., 2016; Hicks et al., 2016). We eventually obtained nectar resource data for 75.7% of all flowering species with nectaries and 89.9% of all flowers recorded in the field survey.

#### 2.2.2 Measurement of pollen volume per flower

To quantify flower‐level pollen volume for each species, we first measured the number of pollen grains per flower and the size and volume of a pollen grain (Hicks et al., 2016). We harvested stamens from randomly selected about‐to‐open buds, or from freshly bloomed flowers for three species whose buds were not available (Supporting Information S1). In total, we collected stamens from an average of nine buds or flowers (range 5–27) from 63 of the 79 species observed in the field survey, as well as 11 species that were not recorded in the survey but appeared outside the observation plots (Table S1). All stamens extracted from an individual flower were stored in a labelled Eppendorf tube suspended in a 0.4 M sucrose solution (30–1000 μL, depending on the size of the samples). We then gently smashed all of the stamens in each tube and shook the tube sufficiently to release all pollen grains into the sucrose solution. Following the method described in Nikkeshi et al. (2019), we mounted 10 μL of the suspension on a glass slide and visually counted the pollen grains included in the solution under a biological microscope (Nikon ECLIPSE E600). We performed this procedure twice and then calculated the mean number of pollen grains in a 10‐μL extract of the solution and the total number of pollen grains per flower based on the initial solution volume. The mean number of pollen grains per flower was then obtained for each species. We also measured the lengths of the major and minor axes of a pollen grain (μm) on randomly selected pollen grains of each species (range 8–60 grains) under the microscope and calculated the mean lengths of the major and minor axes of the pollen grains for each species. The species mean volume of a pollen grain (μm^3^) was calculated as *V* = 4/3π*AB* ^2^, where *A* is half of the mean length of the major axis of the pollen grain, and *B* is half of the mean length of the minor axis (Hicks et al., 2016). For each species, flower‐level pollen volume (μL) was then obtained by multiplying the mean number of pollen grains per flower by the mean volume of a pollen grain. We obtained pollen resource data for 79.7% of all species and 96.9% of all flowers observed in the field.

#### 2.2.3 Area‐based resource availability

For each species recorded in the survey plots and subjected to resource measurements, we calculated its nectar sugar mass and pollen volume per 1 m^2^ plot area for each observation occasion by multiplying the recorded floral abundance and mean value of flower‐level nectar sugar mass or pollen volume. The total floral resources per plot for each survey occasion were obtained as the sum of nectar sugar mass or pollen volume across species observed in the plot at the time.

#### 2.2.4 Calculating flowering species contributions to resource provision and insect visits

Because the number of flowering species in a single plot was limited and did not capture the whole flowering community composition at a site, we calculated species contributions to resource provision monthly at a river scale, pooling the data from all the plots, as the proportion of nectar sugar mass and pollen volume provided by each plant species in the total amount of these resources provided at each river site in every study month. Additionally, to investigate which plant species were most frequently visited by insects in relative terms, we calculated the proportion of bee and wasp/hoverfly/butterfly/all insect visits recorded for each flowering species to the total numbers of bee and wasp/hoverfly/butterfly/all insect visits observed at each river site every study month. Finally, annual contributions of each plant species to gross floral resource provision and insect visits were calculated for each river site separately for the first year and second year of the study.

### 2.3 Statistical analysis

#### 2.3.1 Seasonal trend in resource supply and insect visits

To model the seasonal fluctuations in floral resource supply and insect visits during the study years, we used generalised additive models (GAMs), which allowed us to illustrate nonlinear trends in these variables over time. For sugar mass and pollen volume, which were incorporated into the models as (sugar mass or pollen volume + 0.0001) as in Timberlake et al. (2019), a gamma error distribution with a log link function was applied to address positive continuous data including zero values. For insect visits, we applied a negative binomial distribution with a log link function to address overdispersed count data. A separate model was constructed for each insect group, that is, bees and wasps, hoverflies and butterflies, and for overall taxa with pooled groups. The theta parameter of each negative binomial distribution was estimated during model fitting. All the analyses described above were conducted separately for each river site.

#### 2.3.2 Diversity of floral resources and insect‐visited flowers

To obtain statistical measures of diversity in flowers contributing to resource supply and of flowers visited by wild insects on a yearly basis, we calculated Shannon's diversity index (*H*′), which considers both species richness (*S*) and evenness of proportional abundance of each species (*J'*). We expected that if some alien plant species predominantly contributed to gross resource provision and were most frequently visited by wild insects throughout the year (i.e. supporting the first hypothesis), then the diversity *H*′ of flowers contributing to the resource supply and that of flowers visited by insects would be comparably low. On the other hand, if wild insects did not predominantly visit specific resource‐rich alien plant species while visiting various plant species relatively evenly over the course of the year (i.e. supporting the second hypothesis), then the diversity *H*′ of flowers visited by insects would be higher than that of flowers contributing to resource supply.

For each site, we calculated the diversity *H*′ of the flowers contributing to resource provision and of the flowers visited by insects as follows:

$$H^{\prime }=-\sum_{i=1}^{S}p_{i}\times \ln\;\left(p_{i}\right)$$

where 𝑝_𝑖_ is the proportional contribution of flowering species *i* to the annual total nectar sugar mass or pollen volume or to the annual total bee and wasp/hoverfly/butterfly/all insect visits, and *S* is the total number of flowering species observed by the site. The evenness *J'* was obtained by dividing *H*′ by ln (*S*). We calculated these indices separately for the first and second years of the study. Statistical differences between the diversity *H*′ of the flowers contributing to resource supply and of the flowers visited by wild insects each year were tested using post hoc Tukey multiple comparison tests.

All analyses were conducted using the statistical environment R version 3.6.3 (R Core Team, 2020). The GAMs were fitted using the *mgcv* package (Wood, 2011), diversity indices were calculated using the *vegan* package (Oksanen et al., 2019), and Tukey multiple comparison tests were performed using the *multcomp* package (Hothorn et al., 2008).

## 3 RESULTS

### 3.1 Seasonal trend in resource provision and insect visits

**FIGURE 2:**
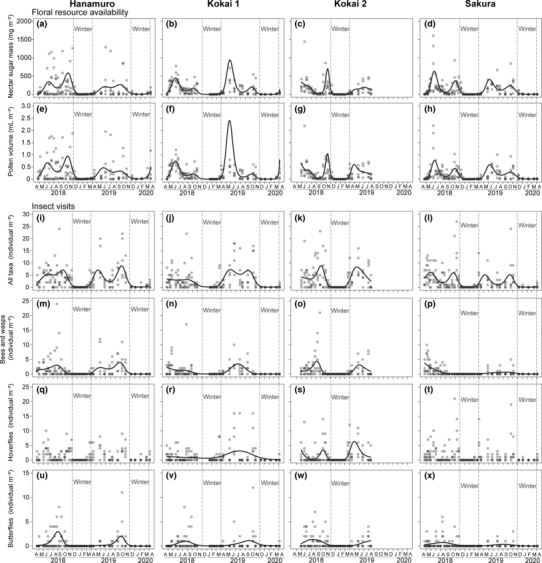
Seasonal patterns in available floral resources (a–d: nectar sugar, e–h: pollen) and insect visits (i–l: overall insect pooling taxa, m–p: wild bees and wasps, q–t: hoverflies and u–x: butterflies) per plot area at the four study sites. The black solid lines indicate GAM predictions (no lines were drawn if statistically significant seasonality was not detected in GAM; for statistics, see Table 1), the grey points represent observed values in each study plot, and the vertical lines delineate the winter period. Note that the grey points are not jittered.

In total, the flowering of 36 alien and 43 native species, each of which showed distinct phenology and floral abundance (Supporting Information S2 and Table S2), was observed during the first and second years of the study. At all sites except for Kokai 1, 5–16 species, including both aliens and natives, co‐flowered at every census during the period from April to November (Supporting Information S2). In Kokai 1, the number of co‐flowering species during the same period occasionally decreased to a minimum of three in the second September. The GAMs demonstrated that the availability of nectar sugar and pollen per plot area significantly fluctuated seasonally at all four sites (Figure 2a–h; Table 1). There was a common seasonal pattern among the four sites in the availability of these resources, where it was high in late spring/early summer (May to July) and autumn (October to November), low in mid‐summer (August to September) and close to zero during winter (December to March).

**TABLE 1:** Summary of the results of generalised additive models (GAMs) examining seasonal fluctuations in floral resource availability and insect visits per plot area.

Model	Parameter	Est. df (estimate)	Ref. df (SE)	Statistical value	*p* value	Deviance explained%
Hanamuro						
Nectar sugar mass	Intercept	(11.091)	(0.138)	80.17	<.001	26.3
	s (census day)	8.822	8.991	16.73	<.001	
Pollen volume	Intercept	(4.885)	(0.144)	34.0	<.001	25.6
	s (census day)	8.841	8.992	14.09	<.001	
All insect visits	Intercept	(0.422)	(0.118)	3.567	<.001	49.9
	s (census day)	8.723	8.975	109.7	<.001	
Bee and wasp visits	Intercept	(−1.935)	(0.699)	−2.767	.006	51.9
	s (census day)	7.419	8.014	31.61	<.001	
Hoverfly visits	Intercept	(0.276)	(0.133)	2.091	.037	0.3
	s (census day)	1.001	1.001	0.536	.465	
Butterfly visits	Intercept	(−3.227)	(0.950)	−3.398	<.001	58.3
	s (census day)	7.002	7.733	32.03	<.001	
Kokai 1						
Nectar sugar mass	Intercept	(10.403)	(0.089)	116.5	<.001	44.5
	s (census day)	8.934	8.999	96.01	<.001	
Pollen volume	Intercept	(4.194)	(0.093)	45.25	<.001	47
	s (census day)	8.945	8.999	76.07	<.001	
All insect visits	Intercept	(0.354)	(0.137)	2.589	.01	40.4
	s (census day)	8.247	8.832	68.26	<.001	
Bee and wasp visits	Intercept	(−1.812)	(0.712)	−2.545	.011	46.5
	s (census day)	6.212	7.11	37.66	<.001	
Hoverfly visits	(Intercept)	(−0.043)	(0.171)	−0.254	.799	15.6
	s (census day)	4.459	5.453	16.85	.008	
Butterfly visits	Intercept	(−1.555)	(0.278)	−5.592	<.001	31.7
	s (census day)	6.038	7.178	20.89	.004	
Kokai 2						
Nectar sugar mass	Intercept	(9.938)	(0.135)	73.45	<.001	38.9
	s (census day)	8.744	8.98	65.68	<.001	
Pollen volume	Intercept	(3.685)	(0.129)	28.56	<.001	50.6
	s (census day)	8.851	8.993	76.11	<.001	
All insect visits	Intercept	(0.170)	(0.201)	0.843	.399	51.0
	s (census day)	7.782	8.556	56.62	<.001	
Bee and wasp visits	Intercept	(−1.354)	(0.385)	−3.518	<.001	56.2
	s (census day)	6.741	7.685	38.64	<.001	
Hoverfly visits	Intercept	(−0.683)	(0.264)	−2.583	.01	42.1
	s (census day)	7.54	8.41	37.28	<.001	
Butterfly visits	Intercept	(−1.298)	(0.273)	−4.754	<.001	34.9
	s (census day)	4.966	6.009	24.41	<.001	
Sakura						
Nectar sugar mass	Intercept	(10.420)	(0.114)	91.35	<.001	29.6
	s (census day)	8.906	8.997	45.47	<.001	
Pollen volume	Intercept	(3.967)	(0.108)	36.81	<.001	34.7
	s (census day)	8.917	8.998	43.01	<.001	
All insect visits	Intercept	(0.204)	(0.133)	1.531	.126	42.9
	s (census day)	8.7	8.97	85.92	<.001	
Bee and wasp visits	Intercept	(−2.223)	(0.429)	−5.178	<.001	58.6
	s (census day)	6.194	7.208	57.94	<.001	
Hoverfly visits	Intercept	(0.431)	(0.147)	2.929	.003	1.7
	s (census day)	1.111	1.213	3.242	.108	
Butterfly visits	Intercept	(−1.866)	(0.296)	−6.308	<.001	25.8
	s (census day)	5.042	6.14	17.27	.009	

*Note*: The statistical values indicate the *t* value for the intercept and the *F* value for the smooth term, s (census day), of nectar sugar and pollen models and the *Z* value for the intercept and the Chi‐square for the smooth term of insect visit models.

For insect visits, we recorded a total of 1946 individual visits, composed of 675 wild bees and wasps, 953 hoverflies and 318 butterflies throughout the study years. According to the GAM predictions, the abundance of overall wild insect visits essentially corresponded to the seasonal fluctuations in floral resource availability, with two peaks in late spring/early summer and autumn (Figure 2i–l). However, there were differences in the peak timing among the three insect groups (Figure 2m–x), and visits by hoverflies alone in Hanamuro and Sakura did not show statistically significant seasonality (Figure 2q,t; GAMs, *p* > .05, Table 1).

### 3.2 Seasonal and annual contributions of plant species to floral resource provision and insect visits

**FIGURE 3:**
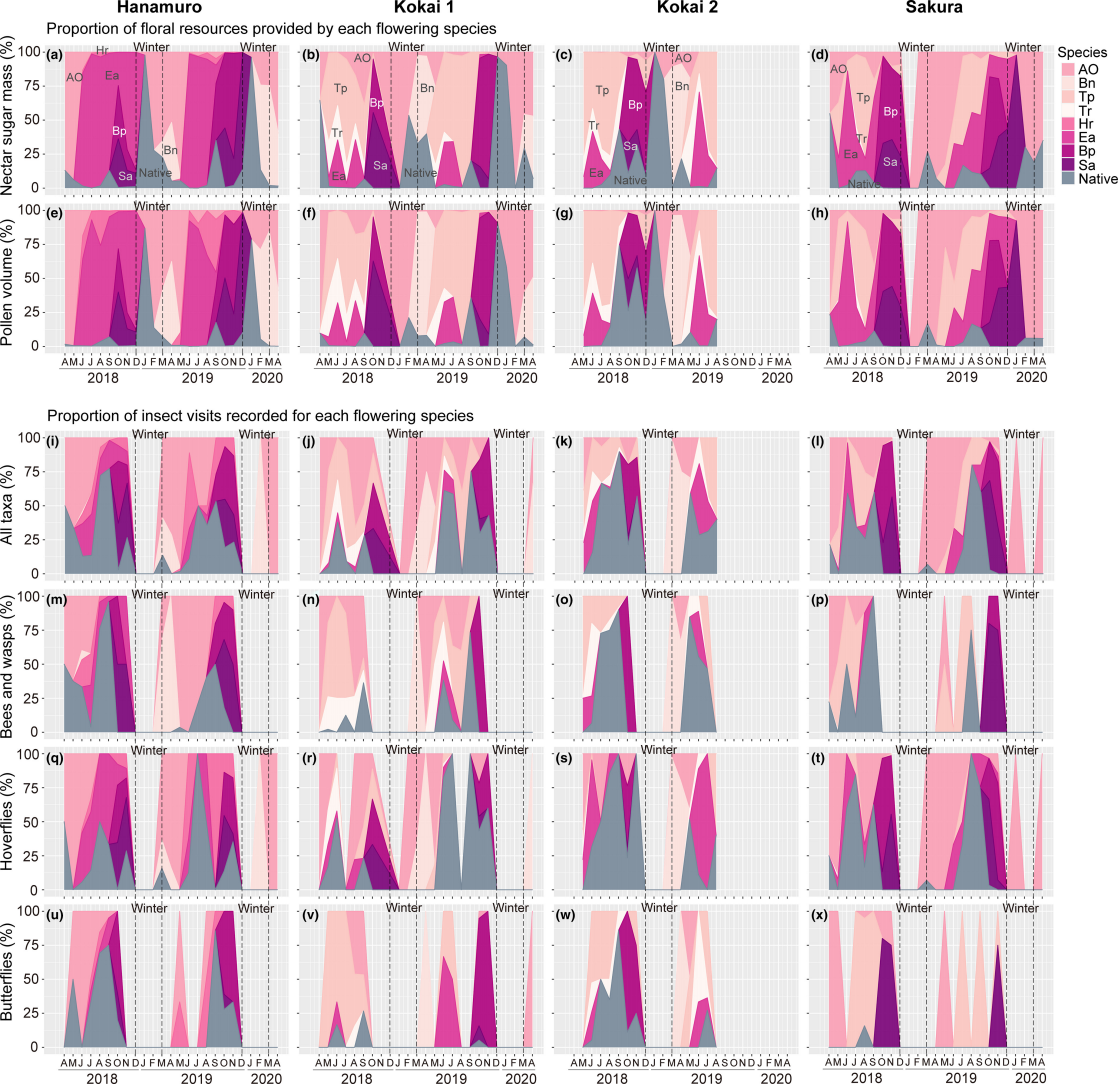
Seasonal patterns in plant species contributions to floral resource provision (a–d: nectar sugar, e–h: pollen) and insect visits (i–l: overall insect pooling taxa, m–p: bees and wasps, q–t: hoverflies and u–x: butterflies) at the four study sites. The vertical lines delineate the winter period. Abbreviations of the species names are Bn: *Brassica napus*, Tp: *Trifolium pratense*, Tr: *T. repens*, Hr: *Hypochaeris radicata*, Ea: *Erigeron annuus*, Bp: *Bidens pilosa* var. *pilosa*, Sa: *Solidago altissima*, AO: all other alien species and Native: all native species (for the last two categories, species identity was pooled due to the small contribution of each species).

At all sites, the available nectar sugar and pollen at the first peak in late spring/early summer (and in mid‐summer at sites other than Kokai 2) were predominantly delivered by either or both of two alien species, *Erigeron annuus* (Asteraceae) and *Trifolium pratense* (Fabaceae), and those in the second peak in autumn were delivered by *Bidens pilosa* var. *pilosa* and *Solidago altissima* (both alien Asteraceae species; Figure 3a–h). At Hanamuro, Kokai 1 and Sakura, the contribution of native species to nectar sugar and pollen provision was continuously low except in winter, when one native (*Lamium amplexicaule*) predominantly flowered (Figure 3a,b,d–f,h). In comparison to at the other three sites, at Kokai 2, the contribution of native species to nectar sugar and pollen provision until winter was higher, especially in September (43% for nectar sugar, 74% for pollen) and November (32% for nectar sugar, 58% for pollen; Figure 3c,g), which was in accordance with the higher native species richness at the site (Supporting Information S2).

The resource‐rich alien plants, *E. annuus*, *T. pratense*, *B. pilosa* var. *pilosa* and *S. altissima*, were visited by all insect groups but not always predominantly. Rather, despite the relatively small contribution to resource provision, native flowering species accounted for high proportions in the visits of all insect groups at all sites except for the case of butterfly visits at Kokai 1 and Sakura (Figure 3i–x). For example, at Hanamuro, the contribution of native flowering species to overall insect visits was as high as 77.0% in the first September (average throughout the period from April to November 28.0%; Figure 2i), even though the native species contribution to resource supply in the same month was less than 15% (average throughout the same period 3.5%; Figure 3a,e).

**FIGURE 4:**
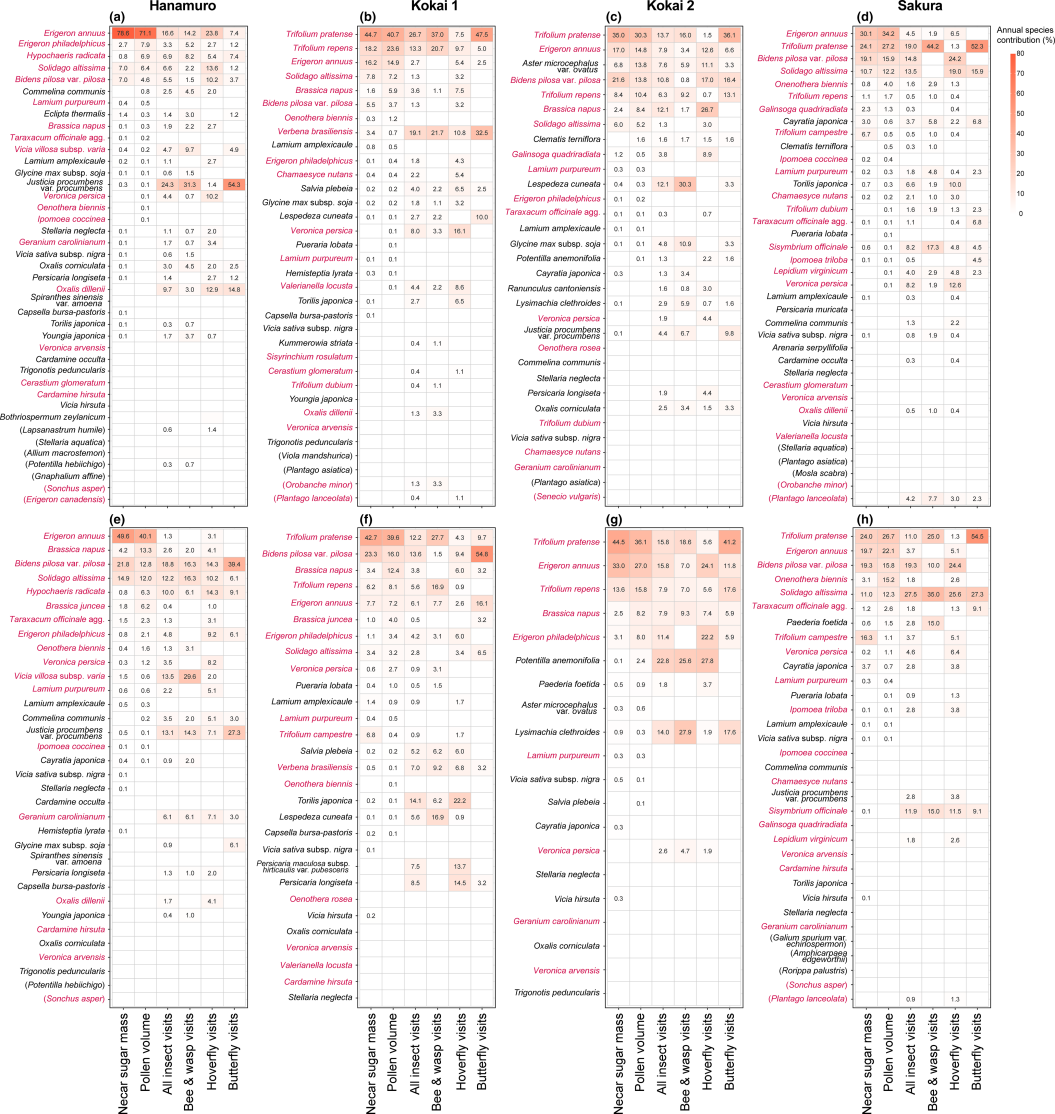
Annual contribution of each plant species to floral resource provision and insect visits at the four river sites in the first (a–d) and second (e–h) years of the study. The value and colour in each tile represent the extent of the contribution; a stronger red colour indicates a higher percentage of the species accounted for in the total amount of provided resources at the site or the total number of insects visiting the sites. White tiles with no values indicate that the contribution of the plant species was less than 0.1%. The order of species names follows the order of the contribution to pollen provision. The colour of the species name represents the origin; pink indicates alien species, and black indicates native species. Species in parentheses are those whose floral resources were not measured.

The annual contributions of each flowering species to nectar sugar and pollen provision and to insect visits also showed distinct patterns (Figure 4a–h). At all sites, either or both of two alien species, *E. annuus* and *T. pratense*, predominantly contributed to the annual resource supply, while wild insects tended to visit more diverse plant species, including various native and alien species, which provided small contributions to resource provision, in both the first and second years at all sites.

### 3.3 Diversity of floral resources and insect‐visited flowers

**FIGURE 5:**
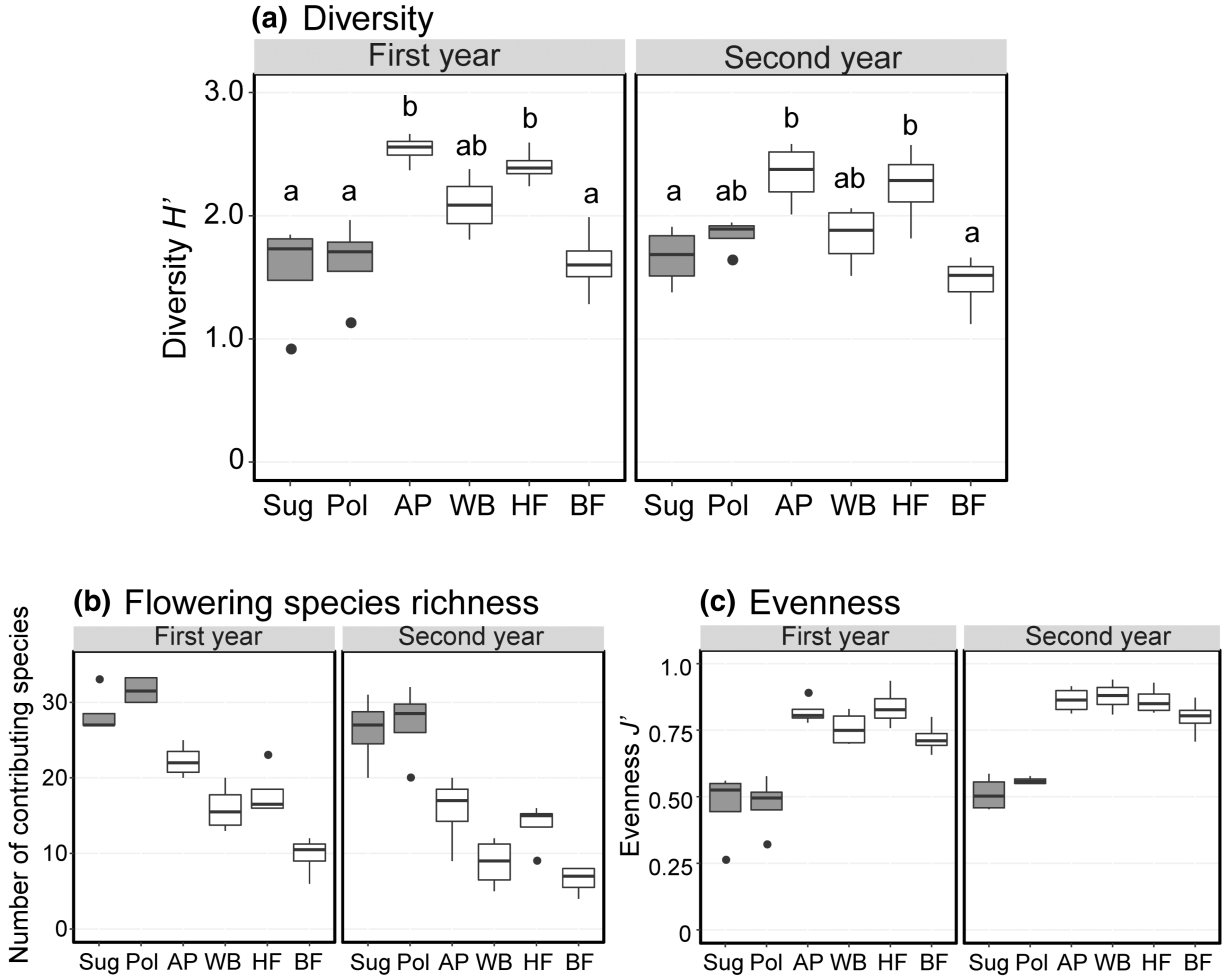
Shannon's diversity index *H*′ (a), richness (b) and evenness *J'* (c) of the flowering species that contributed to floral resource provision (Sug: nectar sugar and Pol: pollen) and of the flowering species visited by insects (AP: overall wild insect pooling taxa, WB: wild bees and wasps, HF: hoverflies and BF: butterflies) in the first and second years of the study. Different letters associated with diversity *H*′ indicate significant differences (Tukey multiple comparison tests, *p* < .05).

Shannon's diversity index *H*′ calculated for flowering species that contributed to the supply of nectar sugar and/or pollen was significantly lower than that calculated for flowering species visited by all wild insects in both study years (Tukey multiple comparison tests, *p* < .05; Figure 5a). Among the three insect groups, flowering species visited by hoverflies showed the highest diversity, followed those visited by bees and wasps, and butterflies. In both years, over 20 flowering species contributed to nectar sugar and pollen supply per site (Figure 5b), but reflecting the uneven species contribution to these resource supplies, the evenness *J'* for resource‐supplying flowering species was less than 0.6 in all cases (Figure 5c). In contrast, the evenness *J'* was much higher for insect‐visited flowering species (Figure 5c). The insects did not visit all the over 20 flowering species that contributed to resource provision, but the number of visited flowering species was larger than that of resource‐rich alien species which predominantly contributed to resource provision (Figure 5b).

## 4 DISCUSSION

To our knowledge, this is the first study to assess the relative contributions of alien and native plant species to nectar sugar and pollen supply and their values to wild pollinating insects in suburban riparian ecosystems. We found that specific alien plants were responsible for the principal part of floral resource provision but were not preferentially visited by wild insects. In the following, we first note the methodological limitations of our approach and then discuss our key findings and the resulting implications for wild pollinator conservation.

### 4.1 Methodological limitations

There are two main limitations in this study. First, as also claimed in previous studies quantifying floral resources (Hicks et al., 2016; Tew et al., 2021), we were forced to follow pragmatic simplification when measuring nectar sugar mass and pollen volume per flower and per unit area. For example, we obtained area‐based nectar sugars of certain species by simply multiplying the recorded floral abundance and mean value of nectar sugar mass of the species, ignoring the variation in the amount of nectar sugar across individual flowers depending on flower age, condition, etc. Additionally, we should note that it was impractical to completely harvest very small amounts of nectar from small flowers, which may have led to underestimations. We carefully validated that our data on nectar sugar mass and pollen volume per flower were consistent with those in previous studies (Supporting Information S1), and we also confirmed that our estimates of floral resources per m^2^ were comparable with the values of perennial meadows published in Hicks et al. (2016). However, due to such methodological issues, we only consider our data as providing relative differences in the order of magnitude of floral resource amount among plant species. Second, to focus on the general responses of overall wild pollinating insects, we did not identify species of flower visitors, which limits the study to some degree. For example, our analyses and discussions did not cover species‐specific interactions, such as relationships between resource‐rich alien plants and specific generalist or specialist pollinator species. Similarly, the differences in floral preferences between native and naturalised alien pollinating insects remain unclarified, although it is unlikely that the latter was substantially included in our insect data because there are only three species of alien pollinating insects recorded in Ibaraki Prefecture (bee: *Bombus terrestris*, hoverfly: *Merodon equestris*, butterfly: *Hestina assimilis*, Ibaraki Prefectural Government, 2022). Species‐specific dependences of wild pollinators on alien floral resources deserve further detailed studies.

### 4.2 Floral resource provision and insect visits in invaded ecosystems

Our year‐round study revealed that in invaded riparian ecosystems at the urban–rural boundary, available nectar sugar and pollen were predominantly derived from a handful of alien plant species throughout the active period of wild flower‐visiting insects. Although butterflies, which reportedly tend to prefer alien plants (Shapiro, 2002), showed a relatively strong preference for resource‐rich alien plants at Kokai 1 and Sakura, the plants were not preferentially visited by wild insects overall. Rather, insects visited various flowering species relatively evenly over the course of the active period, including native species that were not rich in floral resources. The results support the second hypothesis that resource‐rich alien plants do not serve as a principal food source of wild pollinator insects. The limited value of resource‐rich alien plants to wild insects observed here has potential generality, as Williams et al. (2011) also found that wild bees use, but do not particularly prefer, alien plants that are dominant in communities in the grassland–woodland gradients in USA, although the authors did not quantify the floral resources that the alien species actually provided.

The observed pattern of flower visits by wild insects, which was considered more diverse than the floral resource composition based on Shannon's diversity index, may be explained by several mechanisms. First, varied floral preferences of insect species may have caused the pattern. Although we did not identify flower visitors to the species level, it was likely that various species were included in each insect group. Pollinator species have respective preferences for floral traits such as colour, shape and scent (Fründ et al., 2010; Kwaiser & Hendrix, 2008; Szigeti et al., 2023), which are not always associated with the amount of floral resources in flowers (Ortiz et al., 2021). We infer that a substantial portion of insect species included in our samples may have preferred different floral traits from those of the resource‐rich alien species, and therefore, overall, different plant species were more visited. Another possible mechanism is the nutritious advantages of a mixed diet of different floral sources. Plant species differ in the nutrient composition of their floral resources, and a mixed diet containing diverse species resources could offer balanced nutrition (Blüthgen & Klein, 2011; Vaudo et al., 2015). This is the case both for nectar and pollen but possibly with a different extent, given that nectar and pollen provide different nutrition (carbohydrates and protein, respectively) to insects (Ruedenauer et al., 2019). Although most flower‐visiting insects seek both nectar and pollen (Roulston et al., 2000), the degree of qualitative diversity they require may vary between these two resources. Consequently, some insects may have collected nectar and pollen from different flowers, resulting in visitation to diverse flowers overall. Beyond nutritional diversity, a mixed diet of diverse plant species could also reduce the concentration of toxic compounds in the resource acquired from specific plant species, thereby offering safer food (Blüthgen & Klein, 2011; Klaus et al., 2021). To obtain a nutritious and safe diet, wild insects may have sought a variety of floral sources over their activity period, irrespective of relative resource richness, even at the individual insect level. These mechanisms are not mutually exclusive and may have worked simultaneously.

Among the four study river sites, the most resource‐rich flowering species were two aliens, *E. annuus* and *T. pratense*. These species have flower heads rather than a single flower, and the heads supply larger amounts of resources (Hicks et al., 2016). In addition, they bloomed for an exceptionally long period: over 200 days covering the almost entire period that flower visitors were observed (Supporting Information S2). Although the long corolla tubes of *T. pratense* flowers could exclude some small‐sized and/or short‐tongued visitors (Fründ et al., 2010), the floral morphology of *E. annuus* is potentially accessible for various types of pollinating insects, including small hoverflies (Yokoi et al., 2008; Yokoi & Fujisaki, 2009). Despite all these ‘anytime, all you want’ features, on a yearly basis, the alien species received comparable or even fewer floral visits than relatively resource‐poor species, as discussed before. Notably, however, there were moments when insects mainly visited these alien species at all sites (Figure 3i–x). Previous studies have suggested that alien plants with distinct flowering periods can play seasonal roles in providing floral resources for pollinators when the abundance of native floral resources is below the demand (Koyama et al., 2018; Staab et al., 2020). In our study sites, similarly, considering that native flowering species were visited more than would be expected from their contribution to resource provision, it was plausible that some insects might have preferred native plants over aliens, and they visited resource‐rich alien plants only in a particular season when their preferred native flowers were lacking. Nevertheless, the roles of resource‐rich alien species would be considered supplementary, not primary.

### 4.3 Implications for pollinator conservation in suburban ecosystems

The results of our study suggest that the invasion of specific alien plant species due to land development may result in reduced diversity of available floral resources, in contrast to the demand for diverse floral resources from wild pollinator insects. The discrepancy between the diversity of resources required by wild pollinators and that of resources actually available might be common in human‐disturbed ecosystems, given that previous studies on peri‐urban farmland also cautioned that nectar supply was provided by a limited number of species, although they were not aliens, and resource diversity was low (Baude et al., 2016; Timberlake et al., 2019). Resource‐rich flowering species have generally been considered ‘pollinator‐friendly’ and regarded as important for pollinator conservation (Comba et al., 1999; Corbet et al., 2001; Hicks et al., 2016; Tew et al., 2022). Our results that the number of flower visitors corresponded to the peaks of floral resource availability support such positive effects on wild pollinators of increasing the total amount of floral resources by introducing resource‐rich species. However, if there is a trade‐off between the abundance of resource‐rich plants and that of other plants, then the increase in gross floral resources associated with increased abundance of resource‐rich plants would not be beneficial for wild pollinators since this scenario can lead to a decrease in floral resource diversity (Kovács‐Hostyánszki, Szigeti, et al., 2022). Our findings indicate that for the conservation of overall wild pollinating insects in human‐disturbed ecosystems, management that maintains diverse plant species, including natives, would be of great importance, even if it reduces the gross amount of floral resources by suppressing resource‐rich aliens.

## AUTHOR CONTRIBUTIONS

**Chika Egawa:** Conceptualization (lead); formal analysis (lead); funding acquisition (lead); investigation (equal); project administration (lead); visualization (lead); writing – original draft (lead); writing – review and editing (equal). **Teru Yuta:** Investigation (equal); writing – original draft (supporting); writing – review and editing (equal). **Asuka Koyama:** Conceptualization (supporting); investigation (supporting); writing – original draft (supporting); writing – review and editing (equal).

## FUNDING INFORMATION

This research was financially supported by the Japan Society for the Promotion of Science (grant numbers 18K18225, 22K12468).

## CONFLICT OF INTEREST STATEMENT

The authors have no conflict of interest to declare.

## Supporting information


Supporting information S1

Supporting information S2

Table S1

Table S2

## Data Availability

Data on the amount of nectar sugar and pollen supplied by each flowering species are included in Table S2; data on floral abundance and number of insect visits recorded in the field observations is available in the Zenodo repository (https://doi.org/10.5281/zenodo.7758699).
