# Supplementary material for: Specific alien plant species predominantly deliver nectar sugar and pollen but are not preferentially visited by wild pollinating insects in suburban riparian ecosystems
Source: Ecol Evol. 2023 Aug 22;13(8):e10441. doi: 10.1002/ece3.10441 (PMC10444986; doi:10.1002/ece3.10441)
Supplement: Supplementary file 1 — Supporting information S1 [file ECE3-13-e10441-s004.pdf]

Supporting Information S1. Detailed methods of quantification of floral resources

Basic strategy

Floral resources have generally been quantified in terms of sugar mass and pollen volume (Comba et al. 1999; Torres 2000; Corbet et al. 2001; Baude et al. 2016; Hicks et al. 2016; Nottebrock et al. 2017; Nakamura and Kudo 2019). Sugar mass can be determined based on the volume of nectar and the sugar concentration of the nectar (Corbet et al. 2001; Baude et al. 2016; Hicks et al. 2016), while pollen volume can be determined based on the size and total number of pollen grains (Hicks et al. 2016; Nakamura and Kudo 2019). Accordingly, we first empirically measured the following four variables: the volume of nectar (µl), the sugar concentration (g sucrose/100 g nectar), the number of pollen grains and the major and minor axes of a pollen grain (µm), after which we calculated sugar mass (µg) and pollen volume (µl) based on the measured variables.

The empirical measurement of the variables described above was carried out at the level of a single flower for all except Asteraceae and *Trifolium* of which field observations of floral abundance was conducted at the flower head level (Table A). Following Baude et al. (2016), for species belonging to Asteraceae producing capitula (flower heads), we first measured the resources in a floret and then counted the number of florets per head, and subsequently the species mean amount of resources per head was calculated as the product of the mean number of florets per head and the mean amount of resources in a floret. As an exception, for an Asteraceae species *Youngia japonica*, measurement was conducted direcely at the level of the flower head including all florets in a head (Table S1). For species belonging to *Trifolim* (Fabaceae) that produce densely globose inflorescences (also called flower heads), we counted the number of flowers per inflorescence and measured the resources in a flower. We then quantified the species mean resources per inflorescence following the same procedure

applied for Asteraceae. Exceptions to this strategy were made in *T. campestre* and *T. dubium*, in which the measurement of pollen was conducted at the inflorescence level, as in the case of *Y. japonica* (Table S1).

Table A. Summary of measurement items

	Measurement		Outcomes
	**Common item**	**Additional item**	
Most species	Volume of nectar per flower (μl)<br>The sugar concentration (g sucrose/100 g nectar)	—	Nectar sugar mass per flower (μg)
	The number of pollen grains per flower<br>Major and minor axes of a pollen grain (μm)<br>-> Volume of a pollen grain (μm^3)	—	Pollen volume per flower (μl)
Asteraceae	Volume of nectar per floret (μl)<br>The sugar concentration (g sucrose/100 g nectar)	Number of florets per head (capitulum)	Nectar sugar mass per floret, and per head (μg; for *Y. japonica* only that per head)
	The number of pollen grains per floret<br>Major and minor axes of a pollen grain (μm)<br>-> Volume of a pollen grain (μm^3)		Pollen volume per floret, and per head (μl; for *Y. japonica* only that per head)
Trifolium	Volume of nectar per flower (μl)<br>The sugar concentration (g sucrose/100 g nectar)	Number of flowers per head (inflorescence)	Nectar sugar mass per flower, and per head (μg)
	The number of pollen grains per flower<br>Major and minor axes of a pollen grain (μm)<br>-> Volume of a pollen grain (μm^3)		Pollen volume per flower, and per head (μl; for *T. campeste* and *T. dubium* only that per head)

Sampling of flower samples (flower, floret or flower head) of each species was conducted at the peak flowering time determined based on field observations (for the flowering phenology in the study sites, see Supporting Information S2). For several species with a long flowering period, flowers were collected multiple times during the study period. Where possible, species were sampled in both the first and second years of the study to capture intraspecific variations to as great an extent as possible.

Measurement of nectar sugar mass

Nectar was extracted from randomly selected open but not yet senescing flowers or florets of each species (or flower heads for *Y. japonica*) using 0.25, 0.5, 1.0, 2.0, 5.0 or 10.0 µl microcapillary tubes depending on the size of the samples (total length 41 mm for 10.0 µl tubes and 32 mm for other tubes, Drummond Microcaps, Philadelphia, USA; Figure A). One flower sample was used in one measurement event, but in some cases, multiple samples were used to obtain a measurable amount of nectar, and the amount was divided by the number of flower samples used. In each measurement event, we used as many tubes as necessary to empty the sample(s). Sampling was conducted between 6:00 and 16:30 on rainless days. Prior to sampling, the flowers were bagged with a fine net to avoid insect visits and were allowed to accumulate nectar for 2 h. As written in the main manuscript, this duration of nectar accumulation was shorter than that in previous studies (i.e., 24 h in Baude et al. 2016; Hicks et al. 2016; Tew et al. 2021) and would not be appropriate for assessing absolute nectar productivity of species; however, because our focus was on capturing the relative differences among species in contribution to available floral resources, we considered that this accumulation duration can be justified for this study.

We determined the nectar volume by measuring the length of the microcapillary tube occupied by the extracted nectar. The sugar concentration of the sampled nectar was then immediately measured using a hand-held sucrose refractometer modified for small sample volumes (Bellingham and Stanley Eclipse 45-81 and 45-82, Tunbridge Wells, UK; Figure A). For some species producing small flowers/florets, we applied the procedure described by Baude et al. (2016) and Hicks et al. (2016); first, we rinsed a flower or floret with 2 µl of distilled water applied at the location of the nectaries using a micropipette and then measured the sugar concentration of the resulting solution after waiting for 1 min (for the list of species, see Table S1). Because sugar concentration measurements are temperature dependent, we

calibrated the value read with the refractometer to remove the effects of temperature according to the manual provided by the manufacturer (https://www.scientificlabs.co.uk/handlers/libraryFiles.ashx?filename=Manuals_R_REF1050_C.pdf, last accessed on 30 May 2021). We adopted the calibrated sugar concentration value to calculate sugar mass.

Following previous studies (Baude et al. 2016; Hicks et al. 2016), we calculated the sugar mass per flower or floret, or per flower head for *Y. japonica* (s; μg), with the equation $s = 10dv\mathrm{C}$, where v is the volume of nectar measured as indicated above (μl), and d is the density of a sucrose solution with a concentration of C (g sucrose/100 g solution) as obtained from the refractometer. The density of the sucrose solution was calculated as $d = 0.0037921\mathrm{C} + 0.0000178\mathrm{C}^2 + 0.9988603$ (Baude et al. 2016; Hicks et al. 2016). Sugar mass was calculated for each of the collected flower samples, and the mean value for the species was then determined.

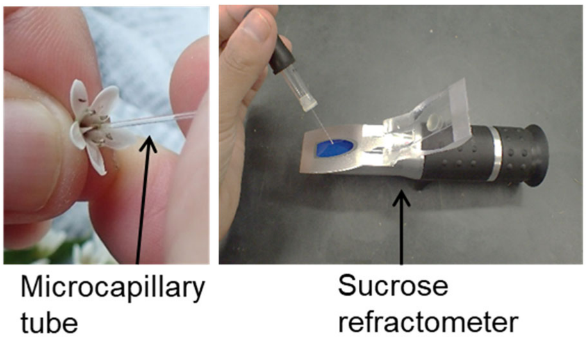

Figure A. Equipment for the nectar measurement

Measurement of pollen volume

To count the number of pollen grains, stamens were harvested from randomly selected about-to-open buds of the flowers or florets of each species (or unopened flower heads for *Y. japonica*, *T. campestre* and *T. dubium*). For *Lotus corniculatus* var. *corniculatus* (Fabaceae), *Sisyrinchium rosulatum* (Iridaceae) and *Ranunculus cantoniensis* (Ranunculaceae), we collected stamens from freshly bloomed flowers instead of buds due to the unavailability of

buds. All stamens extracted from an individual flower sample were stored in a labeled 1.5 ml Eppendorf tube suspended in a 0.4 M sucrose solution (30–1000 µl, depending on the size of the samples). Occasionally, stamens extracted from multiple flower samples were pooled and stored in a tube together to obtain a countable number of pollen grains. We then gently smashed all of the stamens in each tube and shook the tube sufficiently to release all pollen into the sucrose solution. Following Nikkeshi et al. (2019), we mounted 10 µl of the suspension on a glass slide and visually counted the pollen grains included in the solution under a biological microscope (Nikon ECLIPSE E600, Tokyo, Japan). This procedure was performed twice, and we then calculated the mean number of pollen grains in a 10-µl extract of the solution and the total number of grains included in a tube based on the initial solution volume. Finally, we obtained the number of pollen grains per flower/floret/head by dividing the total number of grains by the number of flower samples in the tube.

The lengths of the major and minor axes of a pollen grain (Figure B) were measured on randomly selected pollen grains of each species (range 8–60 grains) under the microscope.

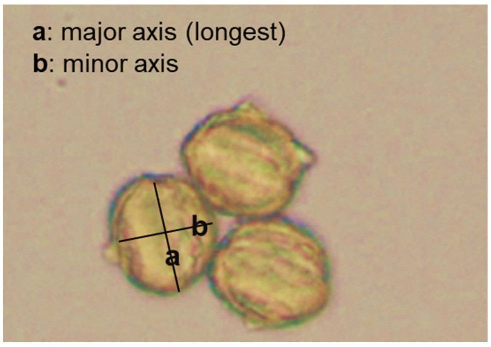

Figure B. Major (a) and minor (b) axes of a pollen grain

For each species, we calculated the mean lengths of the major and minor axes of the pollen grains and the mean number of pollen grains per flower/floret/head. The species mean volume of a pollen grain (μm^3) was calculated as $V = 4/3\pi AB^2$, where A is half of the mean length of the major axis of the pollen grain, and B is half of the mean length of the minor axis (Hicks et al. 2006). The species mean pollen volume (µl) per flower or floret (or per flower head for *Y. japonica*, *T. campestre* and *T. dubium*) was then obtained by multiplying the mean

number of pollen grains per flower/floret/head by the mean volume of a pollen grain.

Data verification

The validity of the species mean values of nectar sugar mass and pollen volume calculated in this study was assessed by comparing our data with the data from previously published studies. We performed standardised major axis linear regression (sma function in the ”smart” library) using the statistical package R ver. 3.6.3 (R Core Team 2020). For the data on the sugar mass per flower or floret in units of µg, we compared the species mean values of 13 shared species across six families between our study and the study by Baude et al. (2016) (for the list of species used for validation, see Table S1). There was a significant positive correlation between the two datasets with a slope that was reasonably close to 1 (slope = 0.63, intercept = 7.86, $R^2 = 0.93$, $P < 0.001$; Figure C (a)). As shown by the slope of 0.63, our data were slightly smaller than that in Baude et al. (2016); this was reasonable because the duration of nectar accumulation we applied was shorter than Baude et al. (2016). This result indicates that our sugar mass values were consistent with the published values. For the pollen volume per flower or floret in units of µl, we compared our species mean values with those of Hicks et al. (2016). Because of the limited number of shared species (eight species across five families; for the list of species used for validation, see Table S1), the correlation was not significant (slope = 1.67, intercept = 0.03, $R^2 = 0.46$, $P = 0.066$), and the uncertainty was large (Figure C (b)). However, some species showed similar pollen volumes in the two studies; for example, 0.005 µl and 0.007 µl for *Sisymbrium officinale*, 0.184 µl and 0.146 µl for *Lotus corniculatus*, and 0.007 µl and 0.003 µl for *Persicaria maculosa* in our study and that of Hicks et al. (2016), respectively. These results support potential concordance between the two datasets.

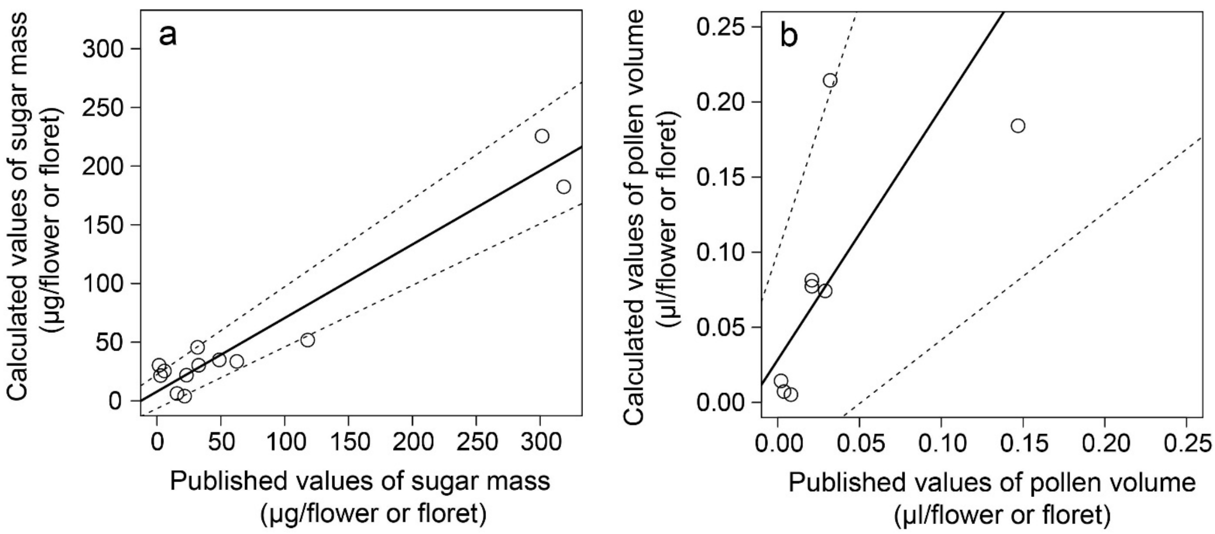

Figure C. Results of data verification using standardised major axis linear regression. (a) Comparison of the species mean sugar mass per flower or floret obtained in this study (y axis) with that in Baude et al. (2016) (x axis) and (b) comparison of the species mean pollen volume per flower or floret calculated in this study (y axis) with that in Hicks et al. (2016) (x axis). Open circles indicate observed values for the species, and solid and dashed lines indicate the estimated values and 95% confidence intervals, respectively.

References

Baude, M., Kunin, W. E., Boatman, N. D., Conyers, S., Davies, N., Gillespie, M. A. K., Morton, R. D., Smart, S. M., & Memmott, J. (2016). Historical nectar assessment reveals the fall and rise of floral resources in Britain. Nature, 530, 85– 88. https://doi.org/10.1038/natur e16532

Comba, L., Corbet, S. A., Hunt, L., & Warren, B. (1999). Flowers, nectar and insect visits: Evaluating British plant species for pollinator-friendly gardens. Annals of Botany, 83, 369–383. https://doi.org/10.1006/anbo.1998.0835

Corbet, S. A., Bee, J., Dasmahapatra, K., Gale, S., Gorringe, E., La Ferla, B., Moorhouse, T., Trevail, A., Van Bergen, Y., & Vorontsova, M. (2001). Native or exotic? Double or single? Evaluating plants for pollinator- friendly gardens. Annals of Botany, 87, 219–232. https://doi.org/10.1006/anbo.2000.1322

Hicks, D. M., Ouvrard, P., Baldock, K. C. R., Baude, M., Goddard, M. A., Kunin, W. E., Mitschunas, N., Memmott, J., Morse, H., Nikolitsi, M., Osgathorpe, L. M., Potts, S. G., Robertson, K. M., Scott, A. V., Sinclair, F., Westbury, D. B., & Stone, G. N. (2016). Food for pollina- tors: Quantifying the nectar and pollen resources of urban flower meadows. PLoS ONE, 11, e0158117. https://doi.org/10.1371/journ al. pone.0158117

Nakamura, S., & Kudo, G. (2019). The influence of garden flowers on pollinator visits to forest flowers: comparison of bumblebee habitat use between urban and natural areas. Urban Ecosystems, 22, 1097–1112. https://doi.org/10.1007/s11252-019-00891-5

Nikkeshi, A., Inoue, H., Arai, T., Kishi, S., & Kamo, T. (2019). The bumblebee *Bombus*

ardens ardens (Hymenoptera: Apidae) is the most important pollinator of Oriental persimmon, *Diospyros kaki* (Ericales: Ebenaceae), in Hiroshima, Japan. Applied Entomology and Zoology, 54, 409–419. https://doi.org/10.1007/s13355-019-00637-x

Nottebrock, H., Schmid, B., Mayer, K., Devaux, C., Esler, K. J., Böhning-Gaese, K., Schleuning, M., Pagel, J. & Schurr, F. M. (2017). Sugar landscapes and pollinator-mediated interactions in plant communities. Ecography, 40, 1129–1138. https://doi.org/10.1111/ecog.02441

R Core Team. (2020) R: A language and environment for statistical computing. R Foundation for Statistical Computing, Vienna, Austria. https://www.R-project.org/

Tew, N. E., Memmott, J., Vaughan, I. P., Bird, S., Stone, G. N., Potts, S. G., & Baldock, K. C. R. (2021). Quantifying nectar production by flowering plants in urban and rural landscapes. Journal of Ecology, 109, 1747–1757. https://doi.org/10.1111/1365-2745.13598

Torres, C. (2000). Pollen size evolution: Correlation between pollen volume and pistil length in Asteraceae. Sexual Plant Reproduction, 12, 365–370. https://doi.org/10.1007/s004970000030
