# Supplementary material for: Specific alien plant species predominantly deliver nectar sugar and pollen but are not preferentially visited by wild pollinating insects in suburban riparian ecosystems
Source: Ecol Evol. 2023 Aug 22;13(8):e10441. doi: 10.1002/ece3.10441 (PMC10444986; doi:10.1002/ece3.10441)
Supplement: Supplementary file 2 — Supporting information S2 [file ECE3-13-e10441-s002.pdf]

# Supporting Information S2. Flowering phenology

## Hanamuro

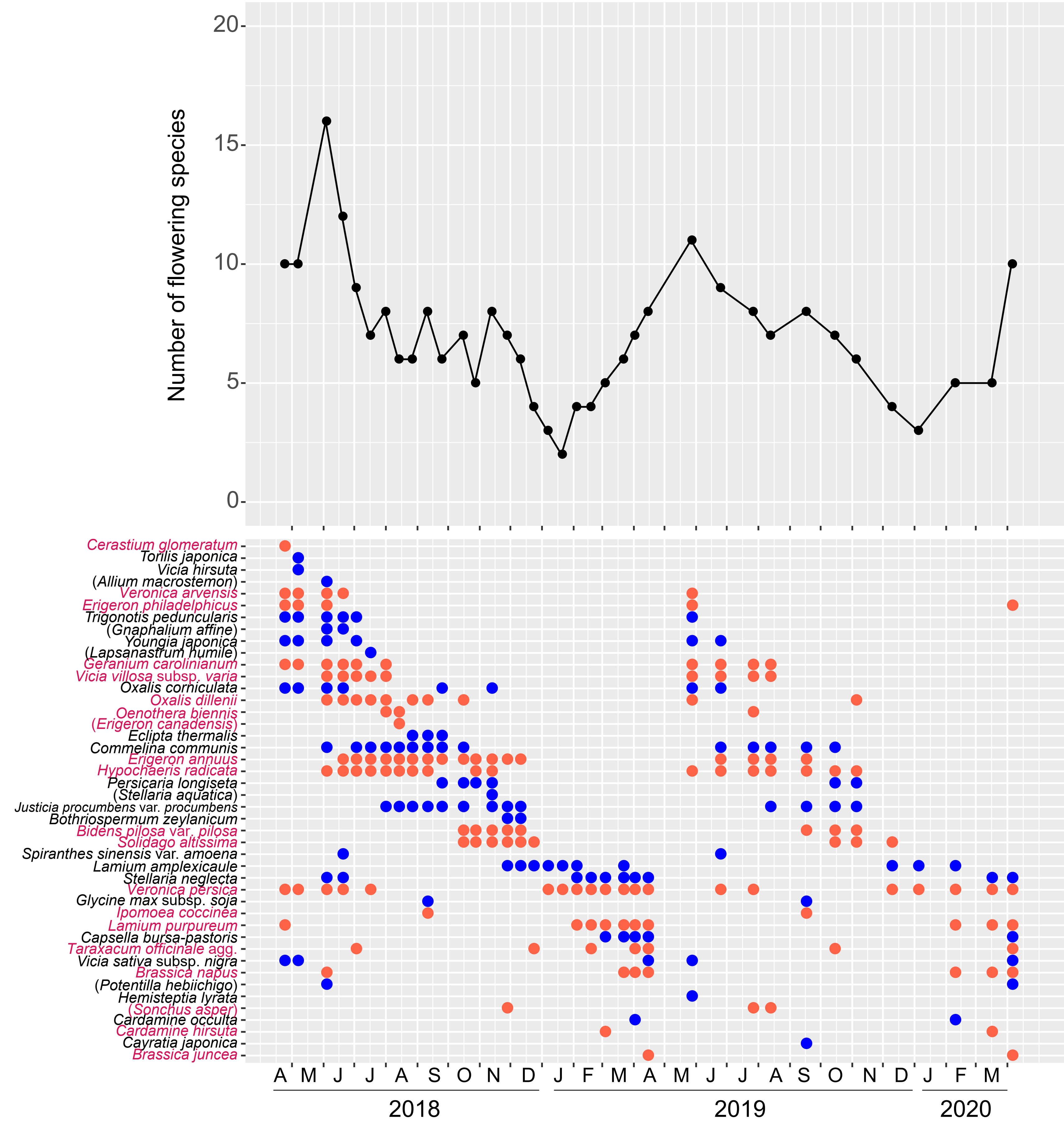


The colour of the species name represents the origin; pink indicates alien species and black indicates native species.
Species in parentheses are those whose floral resources were not measured.

# Supporting Information S2. Flowering phenology

## Kokai 1

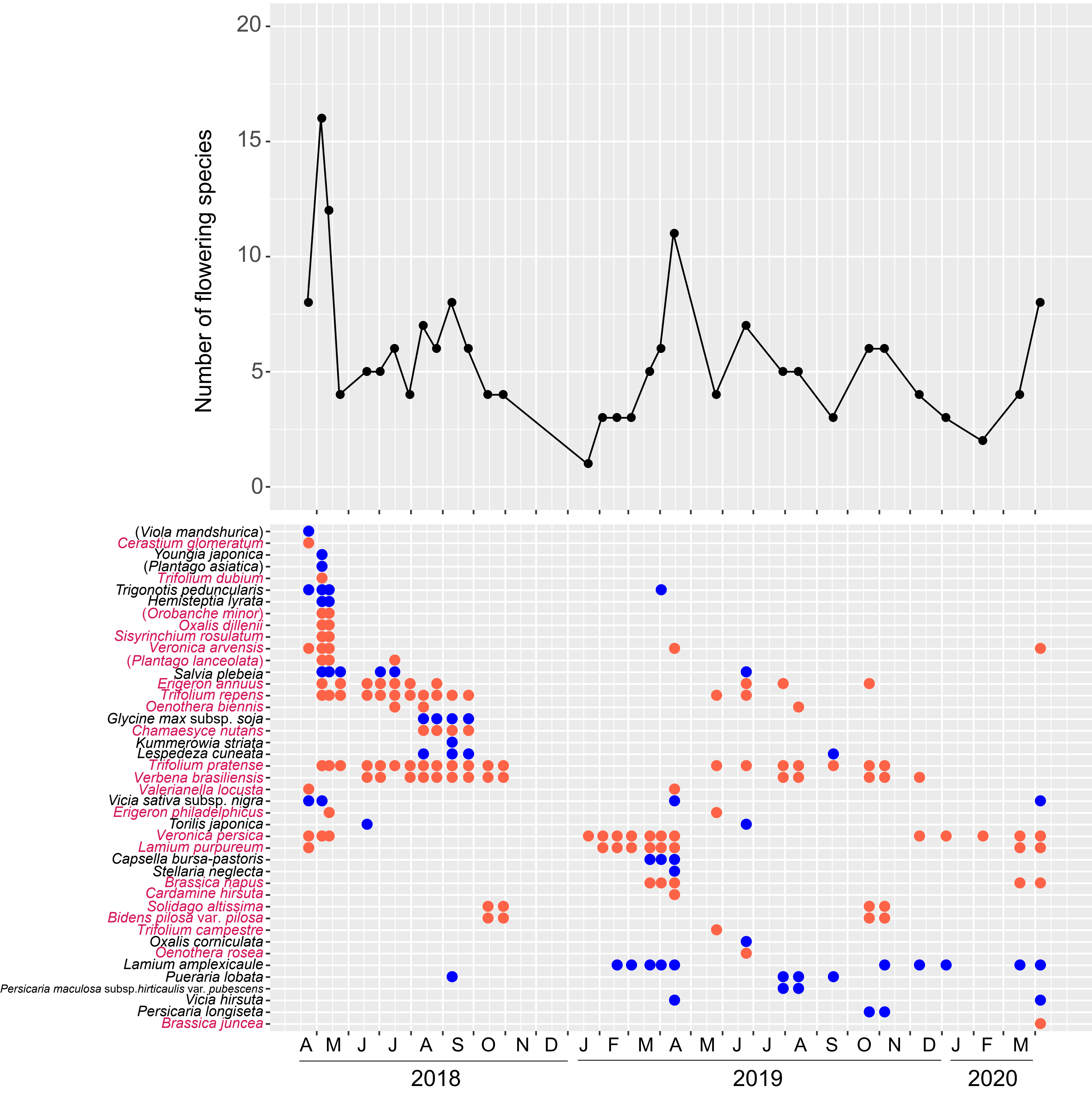


The colour of the species name represents the origin; pink indicates alien species and black indicates native species.
Species in parentheses are those whose floral resources were not measured.

## Supporting Information S2. Flowering phenology

### Kokai 2

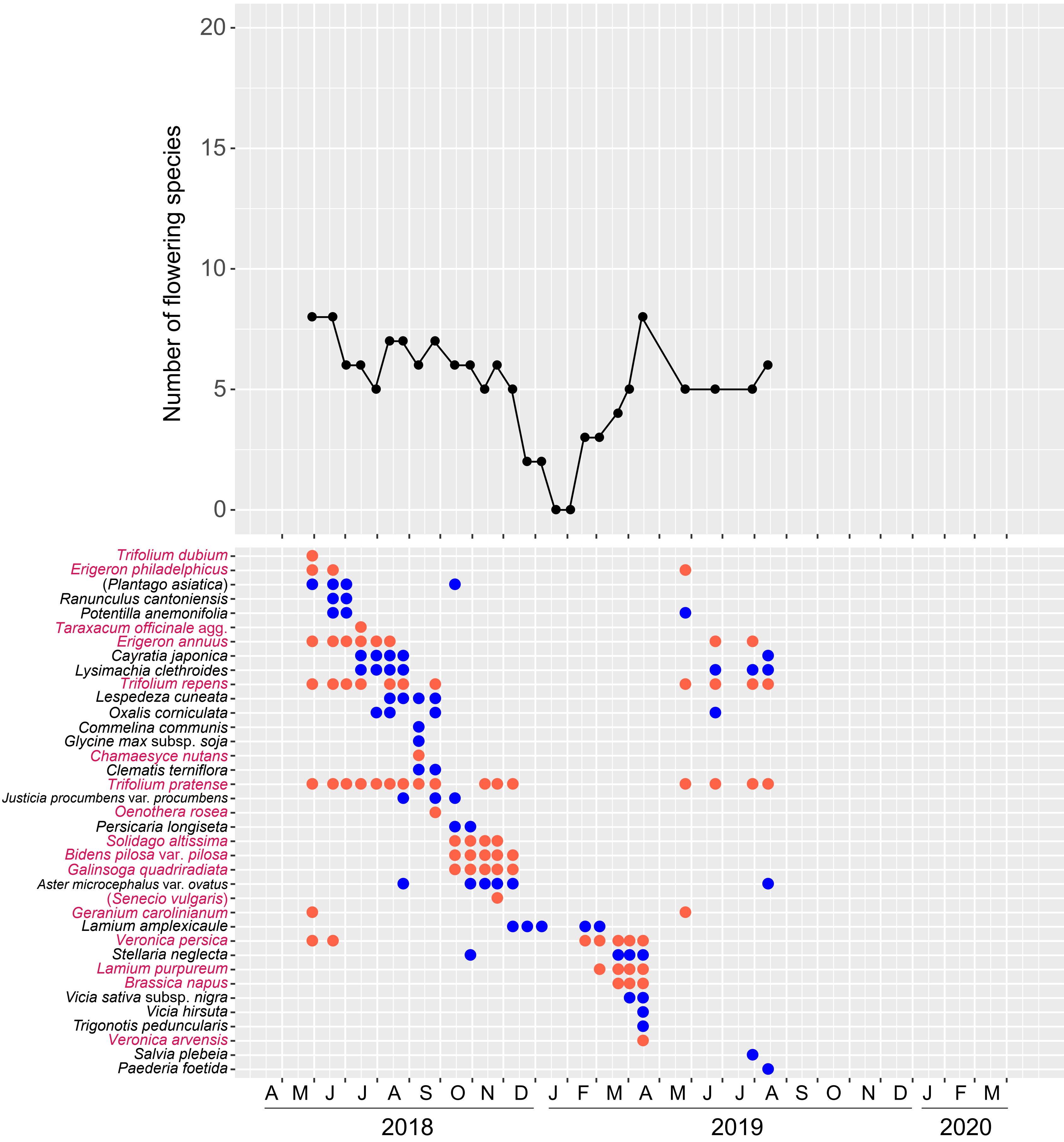


The colour of the species name represents the origin; pink indicates alien species and black indicates native species.
Species in parentheses are those whose floral resources were not measured.

# Supporting Information S2. Flowering phenology
## Sakura

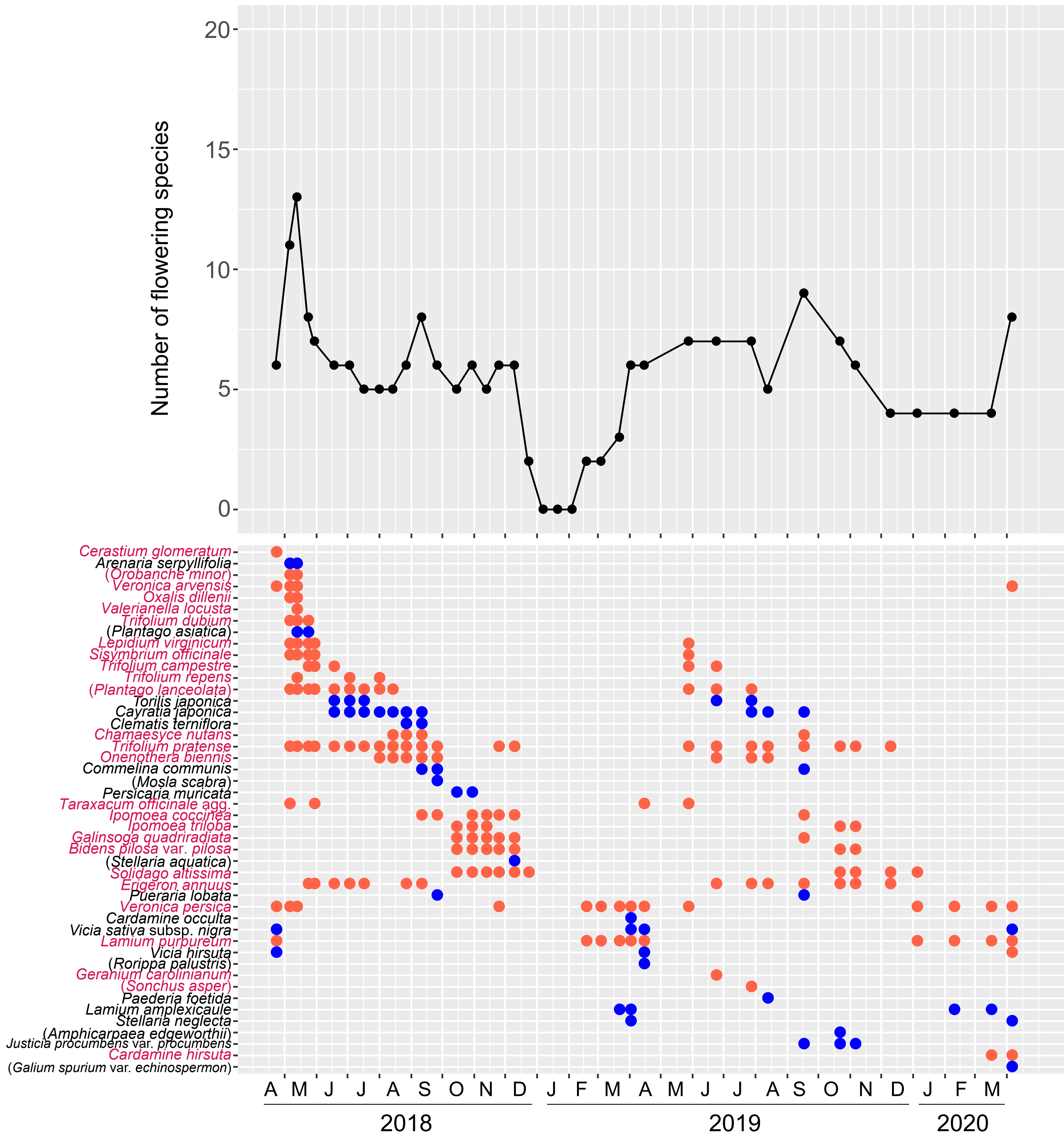


The colour of the species name represents the origin; pink indicates alien species and black indicates native species.
Species in parentheses are those whose floral resources were not measured.

## Supporting Information S2. Flowering phenology

## Summary of flowering phenology of all recorded species (site pooled)

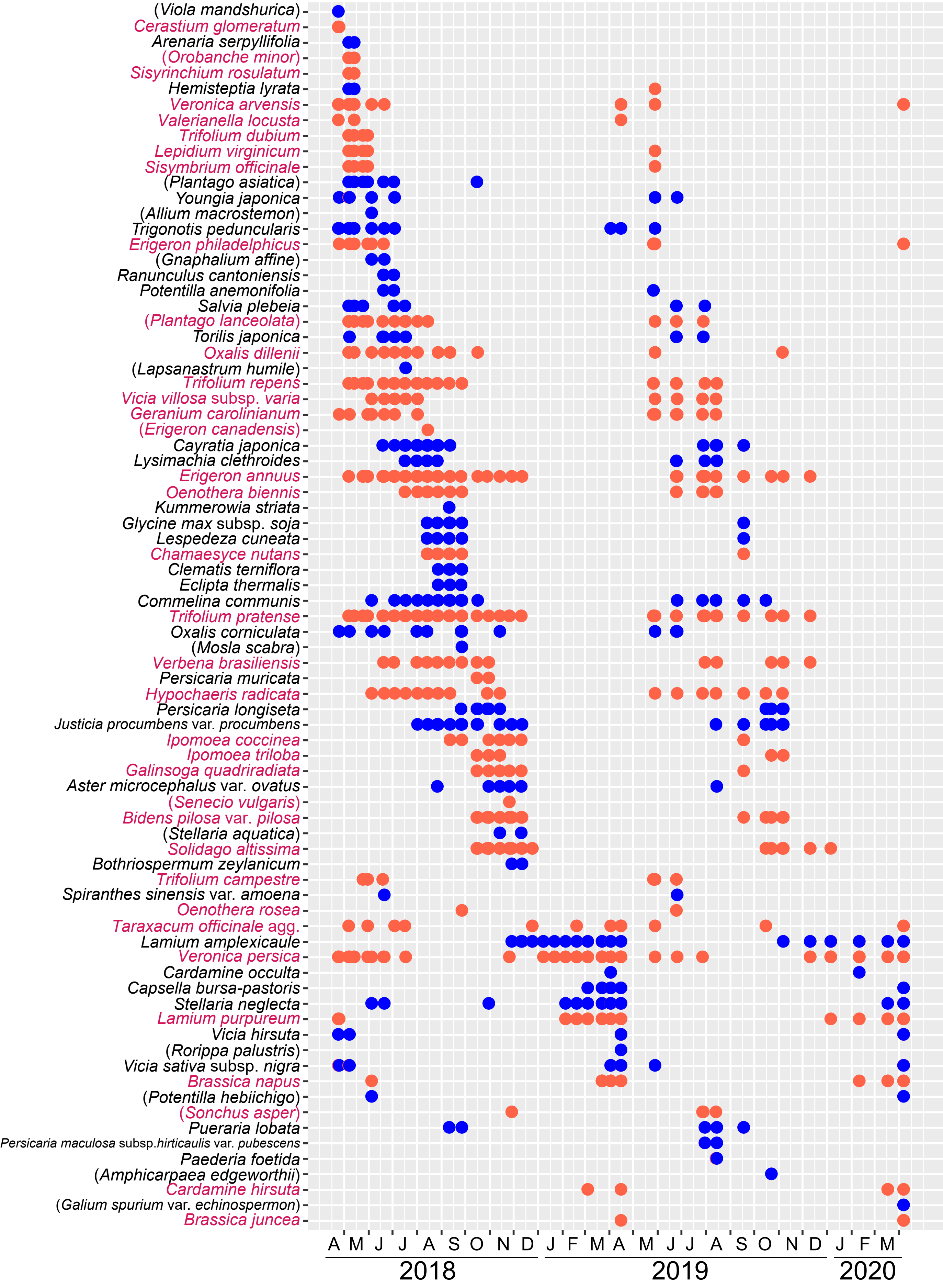


The colour of the species name represents the origin; pink indicates alien species and black indicates native species. Species in parentheses are those whose floral resources were not measured.
