# Supplementary material for: Specific alien plant species predominantly deliver nectar sugar and pollen but are not preferentially visited by wild pollinating insects in suburban riparian ecosystems
Source: Ecol Evol. 2023 Aug 22;13(8):e10441. doi: 10.1002/ece3.10441 (PMC10444986; doi:10.1002/ece3.10441)
Supplement: Supplementary file 3 — Table S1 [file ECE3-13-e10441-s003.pdf]

Table S1. Overview of studied species. Species nomenclature was based on Ylist (http://ylist.info, last accessed on 22 March 2023).

Family	Species	Origin	Nectar measurement unit	Nectar sample N	Sugar concentration measurement [a]	Pollen measurement unit	Pollen sample N	Validation [b]	Notes
**Flowering species recorded in the survey plots and subjected to resource measurements**									
Acanthaceae	*Justicia procumbens* var. *procumbens*	Native	Flower	23	Direct	Flower	9		
Apiaceae	*Torilis japonica*	Native	Flower	26	Direct	Flower	20		
Asteraceae	*Aster microcephalus* var. *ovatus*	Native	Floret	25	Direct	Floret	14		Resource per flower head (floral unit) was then calculated [c]
	Bidens pilosa var. *pilosa*	Alien	Floret	25	Direct	Floret	13		Resource per flower head (floral unit) was then calculated [c]
	Eclipta thermalis	Native	Floret	6	Rinse	Floret	6		Resource per flower head (floral unit) was then calculated [c]
	Erigeron annuus	Alien	Floret	10	Rinse	Floret	7		Resource per flower head (floral unit) was then calculated [c]
	Erigeron philadelphicus	Alien	Floret	6	Direct	Floret	7		Resource per flower head (floral unit) was then calculated [c]
	Galinsoga quadriradiata	Alien	Floret	5	Rinse	Floret	13		Resource per flower head (floral unit) was then calculated [c]
	Hemisteptia lyrata	Native	Floret	14	Direct	Floret	6		Resource per flower head (floral unit) was then calculated [c]
	Hypochaeris radicata	Alien	Floret	9	Direct	Floret	7	Sugar	Resource per flower head (floral unit) was then calculated[c]
	Solidago altissima	Alien	Floret	28	Direct	Floret	13		Resource per flower head (floral unit) was then calculated [c]
	Taraxacum officinale agg.	Alien	Floret	6	Direct	Floret	6	Sugar	Resource per flower head (floral unit) was then calculated [c]
	Youngia japonica	Native	Flower head	11	Direct	Flower head	5		Measurement was done directly at the level of flower head
Boraginaceae	*Bothriospermum zeylanicum*	Native	Flower	5	Direct	Flower	7		
	Trigonotis peduncularis	Native	Flower	28	Direct	Flower	6		
Brassicaceae	*Brassica juncea*	Alien	Flower	15	Direct	Flower	9		
	Brassica napus	Alien	Flower	16	Direct	Flower	6	Sugar	
	Capsella bursa-pastoris	Native	Flower	10	Rinse	Flower	6	Sugar, Pollen	
	Cardamine hirsuta	Alien	Flower	10	Rinse	Flower	10	Sugar	
	Cardamine occulta	Native	Flower	15	Direct	Flower	8		
	Lepidium virginicum	Alien	NA	NA	NA	Flower	16		Nectar data unavailable due to the very small flower size
	Sisymbrium officinale	Alien	Flower	15	Direct	Flower	5	Sugar, Pollen	
Caryophyllaceae	*Arenaria serpyllifolia*	Native	NA	NA	NA	Flower	6		Nectar data unavailable due to its very small flower size

Table S1. (Continued)

Family	Species	Origin	Nectar measurement unit	Nectar sample N	Sugar concentration measurement [a]	Pollen measurement unit	Pollen sample N	Validation [b]	Notes
Caryophyllaceae	*Cerastium glomeratum*	Alien	Flower	17	Direct	Flower	17		
	Stellaria neglecta	Native	Flower	14	Direct	Flower	9		
Commelinaceae	*Commelina communis*	Native	NA	NA	NA	Flower	7		No nectaries
Convolvulaceae	*Ipomoea coccinea*	Alien	Flower	22	Direct	Flower	27		
	Ipomoea triloba	Alien	Flower	13	Direct	Flower	7		
Euphorbiaceae	*Chamaesyce nutans*	Alien	Flower	12	Direct	Flower	10		
Fabaceae	*Glycine max* subsp. *soja*	Native	Flower	17	Direct	Flower	6		
	Kummerowia striata	Native	Flower	7	Direct	Flower	12		
	Lespedeza cuneata	Native	Flower	13	Direct	Flower	13		
	Pueraria lobata	Native	Flower	19	Direct	Flower	12		
	Trifolium campestre	Alien	Flower	10	Rinse	Flower head	8		
	Trifolium dubium	Alien	NA	NA	NA	Flower head	16		Nectar data unavailable due to the very small flower size
	Trifolium pratense	Alien	Flower	29	Direct	Flower	16	Sugar, Pollen	
	Trifolium repens	Alien	Flower	30	Direct	Flower	16	Sugar, Pollen	
	Vicia hirsuta	Native	Flower	10	Rinse	Flower	6		
	Vicia sativa subsp. *nigra*	Native	Flower	47	Direct	Flower	12	Sugar	
	Vicia villosa subsp. *varia*	Alien	Flower	32	Direct	Flower	11		
Geraniaceae	*Geranium carolinianum*	Alien	Flower	29	Direct	Flower	6		
Iridaceae	*Sisyrinchium rosulatum*	Alien	NA	NA	NA	Flower	5		No nectaries
Lamiaceae	*Lamium amplexicaule*	Native	Flower	38	Direct	Flower	17		
	Lamium purpureum	Alien	Flower	53	Direct	Flower	6	Sugar, Pollen	
Lamiaceae	*Salvia plebeia*	Native	Flower	22	Direct	Flower	14		
Myrsinaceae	*Lysimachia clethroides*	Native	Flower	23	Direct	Flower	13		
Onagraceae	*Oenothera rosea*	Alien	Flower	10	Direct	Flower	6		
	Oenothera biennis	Alien	Flower	9	Direct	Flower	11		
Orchidaceae	*Spiranthes sinensis* var. *amoena*	Native	Flower	31	Direct	Flower	6		
Oxalidaceae	*Oxalis corniculata*	Native	Flower	12	Direct	Flower	6		
	Oxalis dillenii	Alien	Flower	16	Direct	Flower	5		
Plantaginaceae	*Veronica arvensis*	Alien	Flower	10	Rinse	Flower	6		
	Veronica persica	Alien	Flower	49	Direct	Flower	6	Sugar, Pollen	

Table S1. (Continued)

Family	Species	Origin	Nectar measurement unit	Nectar sample N	Sugar concentration measurement [a]	Pollen measurement unit	Pollen sample N	Validation [b]	Notes
Polygonaceae	*Persicaria longiseta*	Native	Flower	11	Direct	Flower	6		
	Persicaria maculosa subsp. *hirticaulis*	Native	Flower	14	Direct	Flower	7	Sugar, Pollen	
	Persicaria muricata	Native	Flower	6	Direct	Flower	7		
Ranunculaceae	*Clematis terniflora*	Native	NA	NA	NA	Flower	5		No nectaries
	Ranunculus cantoniensis	Native	Flower	8	Direct	Flower	9		
Rosaceae	*Potentilla anemonifolia*	Native	Flower	13	Direct	Flower	8		
Rubiaceae	*Paederia foetida*	Native	Flower	18	Direct	Flower	6		
Valerianaceae	*Valerianella locusta*	Alien	NA	NA	NA	Flower	6		Nectar data unavailable due to the very small flower size
Verbenaceae	*Verbena brasiliensis*	Alien	Flower	27	Direct	Flower	17		
Vitaceae	*Cayratia japonica*	Native	Flower	17	Direct	Flower	6		
**Flowering species recorded in the survey plots but not subjected to resource measurements**									
Amaryllidaceae	*Allium macrostemon*	Native							
Asteraceae	*Erigeron canadensis*	Alien							
	Gnaphalium affine	Native							
	Lapsanastrum humile	Native							
	Senecio vulgaris	Alien							
	Sonchus asper	Alien							
Brassicaceae	*Rorippa palustris*	Native							
Caryophyllaceae	*Stellaria aquatica*	Native							
Fabaceae	*Amphicarpaea edgeworthii*	Native							
Lamiaceae	*Mosla scabra*	Native							
Orobanchaceae	*Orobanche minor*	Alien							
Plantaginaceae	*Plantago asiatica*	Native							
	Plantago lanceolata	Alien							
Rosaceae	*Potentilla hebiichigo*	Native							
Rubiaceae	*Galium spurium* var. *echinospermon*	Native							
Violaceae	*Viola mandshurica*	Native							

Table S1. (Continued)

Family	Species	Origin	Nectar measurement unit	Nectar sample N	Sugar concentration measurement [a]	Pollen measurement unit	Pollen sample N	Validation[b]	Notes
**Flowering species not recorded in the survey plots but subjected to resource measurements**									
Amaryllidaceae	*Lycoris radiata*	Native	Flower	10	Direct	Flower	6		
Asteraceae	*Aster yomena* var. *dentatus*	Native	Floret	15	Direct	Floret	7		Resource per flower head (floral unit) was then calculated [c]
Campanulaceae	*Triodanis perfoliata*	Alien	NA	NA	NA	Flower	2		Nectar data unavailable due to the limited number of samples
Cucurbitaceae	*Sicyos angulatus*	Alien	Flower	22	Direct	Flower	6		
Fabaceae	*Chamaecrista nomame*	Native	NA	NA	NA	Flower	5		Nectar data unavailable due to the limited number of samples
	Desmodium paniculatum	Alien	NA	NA	NA	Flower	5		Nectar data unavailable due to the limited number of samples
	Lotus corniculatus var. *corniculatus*	Alien	Flower	5	Direct	Flower	5	Sugar, Pollen	
	Lotus corniculatus var. *japonicus*	Native	NA	NA	NA	Flower	5		Nectar data unavailable due to the limited number of samples
Lamiaceae	*Glechoma hederacea* subsp. *grandis*	Native	Flower	43	Direct	Flower	13		
Polygonaceae	*Persicaria lapathifolia* var. *lapathifolia*	Native	NA	NA	NA	Flower	7		Nectar data unavailable due to the limited number of samples
	Persicaria thunbergii	Native	Flower	14	Direct	Flower	9		

[a] Method applied to measure the sugar concentration in nectar, Direct: nectar was directly dropped from a microcapillary tube onto a sucrose refractometer, Rinse: a flower was rinsed with 2 µl of distilled water, and the resulting solution was dropped onto a refractometer (for details, see Supporting Information S1). [b] Species used for the validation of sugar mass and pollen volume (for details, see Supporting Information S1). [c] For the methods to calculate the amount of resource at a flower head level, see Supporting Information S1.
