# Supplementary material for: Specific alien plant species predominantly deliver nectar sugar and pollen but are not preferentially visited by wild pollinating insects in suburban riparian ecosystems
Source: Ecol Evol. 2023 Aug 22;13(8):e10441. doi: 10.1002/ece3.10441 (PMC10444986; doi:10.1002/ece3.10441)
Supplement: Supplementary file 4 — Table S2 [file ECE3-13-e10441-s001.pdf]

Table S2. Averaged abundance of floral units per plot and resource availability of species recorded in field observations.

Family	Species	Origin	Floral unit	Mean floral density (m^{-1})	Mean nectar sugar mass per floral unit (μg)	Mean pollen volume per floral unit (μL)
Acanthaceae	*Justicia procumbens* var. *procumbens*	Native	Single flower	88	65.165	0.033
Amaryllidaceae	*Allium macrostemon*	Native	Single flower	1	NA	NA
Apiaceae	*Torilis japonica*	Native	Single flower	851	14.168	0.007
Asteraceae	*Aster microcephalus* var. *ovatus*	Native	Flower head	63	1755.78	6.162
	Bidens pilosa var. *pilosa*	Alien	Flower head	75	2823.3	3.12
	Eclipta thermalis	Native	Flower head	48	813.463	0.316
	Erigeron annuus	Alien	Flower head	95	3141.369	4.752
	Erigeron canadensis	Alien	Flower head	22	NA	NA
	Erigeron philadelphicus	Alien	Flower head	35	1109.16	5.4
	Galinsoga quadriradiata	Alien	Flower head	153	254.541	0.186
	Gnaphalium affine	Native	Flower head	1048	NA	NA
	Hemisteptia lyrata	Native	Flower head	2	5776.358	2.254
	Hypochaeris radicata	Alien	Flower head	19	449.18	6.66
	Lapsanastrum humile	Native	Flower head	1	NA	NA
	Senecio vulgaris	Alien	Flower head	4	NA	NA
	Solidago altissima	Alien	Flower head	1570	103.248	0.156
	Sonchus asper	Alien	Flower head	2	NA	NA
	Taraxacum officinale agg.	Alien	Flower head	6	2940.32	8.704
	Youngia japonica	Native	Flower head	18	65.348	0.019
Boraginaceae	*Bothriospermum zeylanicum*	Native	Single flower	111	6.123	< 0.001
	Trigonotis peduncularis	Native	Single flower	4	10.209	0.002
Brassicaceae	*Brassica juncea*	Alien	Single flower	292	166.225	1.089
	Brassica napus	Alien	Single flower	142	182.029	1.086
	Capsella bursa-pastoris	Native	Single flower	113	30.052	0.014
	Cardamine hirsuta	Alien	Single flower	14	25.2	0.008
	Cardamine occulta	Native	Single flower	37	8.242	0.023
	Lepidium virginicum	Alien	Single flower	637	NA	0.002
	Rorippa palustris	Native	Single flower	15	NA	NA
	Sisymbrium officinale	Alien	Single flower	322	21.11	0.005
Caryophyllaceae	*Arenaria serpyllifolia*	Native	Single flower	9	NA	0.013
	Cerastium glomeratum	Alien	Single flower	4	29.756	0.053
	Stellaria aquatica	Native	Single flower	5	NA	NA
	Stellaria neglecta	Native	Single flower	36	19.6	0.016
Commelinaceae	*Commelina communis*	Native	Single flower	16	0 (no nectaries)	0.564
Convolvulaceae	*Ipomoea coccinea*	Alien	Single flower	26	193.953	0.483
	Ipomoea triloba	Alien	Single flower	21	204.924	0.278

Table S2. (Continued)

Family	Species	Origin		Mean floral density (m^{-1})	Mean nectar sugar mass per floral unit (μg)	Mean pollen volume per floral unit (μL)
Euphorbiaceae	*Chamaesyce nutans*	Alien	Single flower	862	9.454	0.014
Fabaceae	*Amphicarpaea edgeworthii*	Native	Single flower	1	NA	NA
	Glycine max subsp. *soja*	Native	Single flower	78	69.309	0.115
	Kummerowia striata	Native	Single flower	99	3.014	0.019
	Lespedeza cuneata	Native	Single flower	193	37.614	0.042
	Pueraria lobata	Native	Single flower	25	202.615	0.861
	Trifolium campestre	Alien	Flower head	100	1231.314	0.115
	Trifolium dubium	Alien	Flower head	57	NA	0.052
	Trifolium pratense	Alien	Flower head	19	5491.989	8.239
	Trifolium repens	Alien	Flower head	30	2181.249	4.662
	Vicia hirsuta	Native	Single flower	34	73.909	0.003
	Vicia sativa subsp. *nigra*	Native	Single flower	8	225.288	0.131
	Vicia villosa subsp. *varia*	Alien	Single flower	97	134.322	0.097
Geraniaceae	*Geranium carolinianum*	Alien	Single flower	8	71.725	0.052
Iridaceae	*Sisyrinchium rosulatum*	Alien	Single flower	7	0 (no nectaries)	0.099
Lamiaceae	*Lamium amplexicaule*	Native	Single flower	69	89.511	0.095
	Lamium purpureum	Alien	Single flower	62	45.186	0.081
	Mosla scabra	Native	Single flower	79	NA	NA
	Salvia plebeia	Native	Single flower	221	16.293	0.038
Myrsinaceae	*Lysimachia clethroides*	Native	Single flower	43	120.087	0.073
Onagraceae	*Oenothera rosea*	Alien	Single flower	2	94.401	1.363
	Oenothera biennis	Alien	Single flower	22	1238.812	8.259
Orchidaceae	*Spiranthes sinensis* var. *amoena*	Native	Single flower	25	97.023	0.077
Orobanchaceae	*Orobanche minor*	Alien	Single flower	94	NA	NA
Oxalidaceae	*Oxalis corniculata*	Native	Single flower	15	57.948	0.036
	Oxalis dillenii	Alien	Single flower	17	15.443	0.018
Plantaginaceae	*Plantago asiatica*	Native	Single flower	2	0 (no nectaries)	NA
	Plantago lanceolata	Alien	Single flower	14	0 (no nectaries)	NA
	Veronica arvensis	Alien	Single flower	12	21.267	0.004
	Veronica persica	Alien	Single flower	14	30.101	0.214

Table S2. (Continued)

Family	Species	Origin		Mean floral density (m^{-1})	Mean nectar sugar mass per floral unit (µg)	Mean pollen volume per floral unit (µL)
Polygonaceae	*Persicaria longiseta*	Native	Single flower	82	18.147	0.012
	Persicaria maculosa subsp. *hirticaulis*	Native	Single flower	198	3.663	0.007
	Persicaria muricata	Native	Single flower	139	9.956	0.027
Ranunculaceae	*Clematis terniflora*	Native	Single flower	134	0 (no nectaries)	0.769
	Ranunculus cantoniensis	Native	Single flower	11	16.437	0.432
Rosaceae	*Potentilla anemonifolia*	Native	Single flower	16	34.965	1.306
	Potentilla hebiichigo	Native	Single flower	7	NA	NA
Rubiaceae	*Galium spurium* var. *echinospermon*	Native	Single flower	91	NA	NA
	Paederia foetida	Native	Single flower	102	292.906	1.011
Valerianaceae	*Valerianella locusta*	Alien	Single flower	1129	NA	0.002
Verbenaceae	*Verbena brasiliensis*	Alien	Single flower	634	36.644	0.012
Vitaceae	*Cayratia japonica*	Native	Single flower	83	365.319	0.095
Violaceae	*Viola mandshurica*	Native	Single flower	3	NA	NA
